# B-Cell-Specific Myd88 L252P Expression Causes a Premalignant Gammopathy Resembling IgM MGUS

**DOI:** 10.3389/fimmu.2020.602868

**Published:** 2020-12-01

**Authors:** Kristin Schmidt, Ulrike Sack, Robin Graf, Wiebke Winkler, Oliver Popp, Philipp Mertins, Thomas Sommermann, Christine Kocks, Klaus Rajewsky

**Affiliations:** ^1^ Immune Regulation and Cancer, Max Delbrück Center for Molecular Medicine in the Helmholtz Association, Berlin, Germany; ^2^ Biology of Malignant Lymphomas, Max Delbrück Center for Molecular Medicine in the Helmholtz Association, Berlin, Germany; ^3^ Proteomics, Max Delbrück Center for Molecular Medicine in the Helmholtz Association, Berlin, Germany; ^4^ Transgenics, Max Delbrück Center for Molecular Medicine in the Helmholtz Association, Berlin, Germany

**Keywords:** monoclonal gammopathy of unknown significance, IgM MGUS, MYD88 L265P mutation, Waldenström’s macroglobulinemia, B cell abnormalities, B cell lymphoma, lymphomagenesis, IgM paraprotein

## Abstract

A highly recurrent somatic L265P mutation in the TIR domain of the signaling adapter MYD88 constitutively activates NF-κB. It occurs in nearly all human patients with Waldenström’s macroglobulinemia (WM), a B cell malignancy caused by IgM-expressing cells. Here, we introduced an inducible leucine to proline point mutation into the mouse Myd88 locus, at the orthologous position L252P. When the mutation was introduced early during B cell development, B cells developed normally. However, IgM-expressing plasma cells accumulated with age in spleen and bone, leading to more than 20-fold elevated serum IgM titers. When introduced into germinal center B cells in the context of an immunization, the Myd88^L252P^ mutation caused prolonged persistence of antigen-specific serum IgM and elevated numbers of antigen-specific IgM plasma cells. Myd88^L252P^-expressing B cells switched normally, but plasma cells expressing other immunoglobulin isotypes did not increase in numbers, implying that IgM expression may be required for the observed cellular expansion. In order to test whether the Myd88^L252P^ mutation can cause clonal expansions, we introduced it into a small fraction of CD19-positive B cells. In this scenario, five out of five mice developed monoclonal IgM serum paraproteins accompanied by an expansion of clonally related plasma cells that expressed mostly hypermutated VDJ regions. Taken together, our data suggest that the Myd88^L252P^ mutation is sufficient to promote aberrant survival and expansion of IgM-expressing plasma cells which in turn can cause IgM monoclonal gammopathy of undetermined significance (MGUS), the premalignant condition that precedes WM.

## Introduction

Waldenström’s macroglobulinemia (WM) is an incurable low-grade lymphoplasmacytic lymphoma, characterized by bone marrow (BM) infiltration of small, IgM-positive lymphocytes with varying degrees of plasmacytoid or plasma cell differentiation and the presence of monoclonal immunoglobulin M (IgM) paraproteins (M-spikes) in the serum (1–5). The great majority of malignant WM cells are monoclonal and carry somatically mutated antibody V region rearrangements, suggesting that transformation occurs at a mature, antigen-experienced B cell stage (6–11).

More than 90% of WM patients harbor a T794C gain-of-function mutation in the myeloid differentiation primary response gene 88 (MYD88), which results in an L265P amino acid substitution in the MYD88 TIR domain (12), promoting an increased propensity for Myd88 oligomerization (13). MYD88 is the canonical adaptor protein for inflammatory signaling pathways downstream of various toll-like receptor (TLR) and interleukin (IL)-1 receptor family members (14). First described in activated B-cell (ABC)-like subtype of diffuse large B-cell lymphoma (DLBCL) [where it occurs in 21% of patients (15)], the MYD88^L265P^ mutation constitutively activates NF-κB and JAK kinase signaling through TLR9, IRAK1 and IRAK4 (16, 17), and independently through BTK (18), conferring a pro-survival advantage to mutated B cells. In line with these findings, an earlier attempt to model the Myd88^L265P^ mutation in mice *in vivo* produced fulminant B lymphoproliferative disease and occasional ABC-DLBCL-type lymphoma (19), while a more recent study reported low-grade lymphoproliferative disease with certain pathological features of WM (20). However, in both mouse models the observed lymphoproliferation was polyclonal.

WM is diagnosed late in life at a median age of 73 years in Caucasians (21). Symptomatic WM is preceded by prolonged asymptomatic phases classified as smoldering (or asymptomatic) WM and IgM monoclonal gammopathy of unknown significance (MGUS) (22–26). With increasingly sensitive methods Myd88^L265P^ mutation could be detected in up to 87% of IgM MGUS patients, suggesting that it is an early event in WM pathogenesis (27–33). A second somatic, highly recurrent genetic event in WM consists of activating C-terminal mutations in the CXCR4 gene, which appear to enhance tumor cell dissemination and survival (34–37) and mostly occur in the context of a mutated Myd88 allele (36, 38, 39). CXCR4 mutations are less frequent (25–40% of WM patients) and probably acquired later during disease progression (36, 38–41).

Consistent with such a scenario, we here present evidence that targeting endogenous expression of the dominant Myd88^L265P^ mutation to a small number of cells in the mouse B cell compartment (at the orthologous position L252P in mouse Myd88) is—by itself—sufficient to cause IgM MGUS, the premalignant condition which precedes WM.

## Material and Methods

### Gene Targeting

The gene targeting strategy was based on the NCBI mouse transcript *NM_010851.2*, where wildtype exons 5 and 6 were flanked with *loxP* sites (4.3kb region). Exons 5 and 6 were duplicated and inserted downstream of the distal *loxP* site followed by an IRES-GFP reporter. The L252P mutation was introduced into the duplicated Exon 5 and a *Neo^R^* marker (flanked by *frt* sites) inserted between wildtype Exon 6 and mutated Exon 5. The targeting vector was generated by amplifying the genomic region of Myd88 using BAC clones from the *C57BL/6J* RPCIB-731 BAC library and subsequent introduction of the point mutation. The linearized targeting vector was co-transfected with sgRNA and a Cas-9-expression vector into the Artemis B6/3 C57BL/6 ES cell line. Targeted clones were isolated using positive (*Neo^R^)* selection and correct integration was verified by Southern blotting. The conditional *Myd88^L252P^* allele was obtained in a germline-transmitting transgenic animal after *in vivo* Flp-mediated removal of the selection markers.

### Cell Culture of B Cells *Ex Vivo*

Splenic B cells were enriched by depletion of CD43^+^ cells with magnetic anti-mouse-CD43 microbeads (Miltenyi Biotech Cat# 130-049-801, RRID: AB_2861373), transduced with in-house generated TAT-Cre recombinase (42, 43), cultured in the absence or presence of LPS (20 μg/ml, *Escherichia coli* 055:B5; Sigma Cat# L2880) or F(ab’)_2_ fragment anti-IgM (1.2 μg/ml; Jackson ImmunoResearch Labs Cat# 115-006-020; RRID: AB_2338469) and 1 µM BrdU or cultured with LPS plus recombinant mouse IL-4 (10–20 units/ml; Peprotech Cat# 214-14).

### Flow Cytometry, Cell Sorting, and Detection of *In Vivo* Proliferation

Red blood cells were lysed with Gey’s solution and single-cell suspensions (in PBS pH7.2 supplemented with 1% FCS and 1 mM EDTA) from spleen or femur-derived bone marrow were stained with antibody conjugates (**Supplementary Table 1**) and analyzed using FlowJo software (BD FlowJo, RRID : SCR_008520) on an LSRFortessa (BD Biosciences) or sorted on a FACSAria (BD Biosciences). NIP-BSA-APC: 4-Hydroxy-3-iodo-5-nitrophenylacetyl hapten (NIP) conjugated to Bovine Serum Albumin (BSA) was generated in-house from BSA fraction V (Roth Cat# 8076.3) and NIP-OSu (Biosearch Technologies Cat# N-1080-100) and then labeled with Allophycocyanin (APC) using the Allophycocyanin labeling kit-SH (Dojindo Cat# LK24). For 5-Bromo-2’-deoxyuridine (BrdU) labeling, we used BrdU Kits (BD Biosciences Cat# 552598, RRID: AB_2861367). Mice were injected intraperitoneally with 2 mg BrdU and analyzed by flow cytometry.

### Laboratory Mice and Immunizations

C*γ*1-Cre (44), *R26StopFLeYFP* (45), CD19-Cre (46), and CD19-Cre^ERT2^ alleles (47) have been described. Mice were bred and maintained under specific pathogen-free conditions. Unless specifically indicated (**Supplementary Figures 1B, C**), mice used in this study were heterozygous for the Cre and Myd88^L252P^ alleles (designated Cre;Myd88^L252P^). To activate Cre^ERT2^, four mg of tamoxifen (Sigma Cat# T5648), dissolved in sunflower oil (Sigma Cat# S5007), was fed by oral gavage (47). Eight to 12 weeks old mice were immunized intraperitoneally with 100 µg alum-precipitated 4-Hydroxy-3-nitrophenylacetyl hapten conjugated to Chicken Gamma Globulin (NP-CGG, Ratio 10-19) (LGC Biosearch Technologies Cat# N-5055B-5) followed by secondary immunization intravenously with 100 µg soluble NP-CGG.

### Immunohistochemistry

Tissues were embedded in Tissue-Tek O.C.T. Compound (Sakura Cat# 4583), stored at -80°C and cryosectioned (7 µm thickness). Sections were fixed in 100% acetone and stained with DAPI (eBioScience Cat# D1306), and the antibody conjugates and reagents listed in **Supplementary Table 1**.

### Enzyme-Linked Immuno Assays, Serum Protein Electrophoresis, and Immunofixation

Enzyme-linked immunosorbent assays (ELISAs) were done as described (48) with addition of 0.05% Tween 20 in block and wash buffers. 4-Hydroxy-3-nitrophenylacetyl hapten (NP) conjugated to BSA (NP-BSA, Ratio 28) was generated in-house with BSA fraction V (Roth Cat# 8076.3) and NP-OSu (Biosearch Technologies Cat# N1010-100). Plates were coated with 2 µg/ml NP-BSA or 1 µg/ml anti-light chain antibodies and developed with 1 µg/ml anti-isotype antibodies and the standards listed in **Supplementary Table 1**. For enzyme-linked immuno spot (ELISPOT) assays MultiScreen_HTS_ IP Filter Plates (Merck Cat# MSIPS4510) were coated and developed as described above for the ELISA plates, incubated with cells overnight, washed with 0.1% Tween 20 and processed according to the manufacturer’s instructions. For serum protein electrophoresis or immunofixation 10 µl serum was run on buffered agarose gels, pH8.6 Hydragel PROTEIN(E) (Sebia Cat# PN4100) or pH9.2 DOUBLE IF K20 (Sebia Cat# PN3036), and processed according to the manufacturer’s instructions. For proteomics, serum samples were run on multiple lanes of pH8.6 agarose gels and stained with InstantBlue Ultrafast Protein Stain (Sigma Cat# ISB1L). Excised bands were processed and analyzed by tandem mass spectrometry as described below.

### Sequence Analysis of IgH V Gene Rearrangements

IgH V gene rearrangements were PCR-amplified (40 cycles) from genomic DNA (isolated from sorted, GFP-reporter-positive TACI^+^CD138^+^ plasma cells) using the Expand High Fidelity PCR System (Roche Cat# 03310256103) with a forward primer for J558/VH1 family genes [pos. 37–57 (IMTG) ARG CCT GGG RCT TCA GTG AAG] and a reverse primer for the IgH intronic enhancer (CTCCACCAGACCTCTCTAGACAGC). A 0.9 kb fragment corresponding to JH4 rearrangements was gel-purified, cloned (Zero Blunt TOPO PCR Cloning Kit, Invitrogen Cat# 450031) and subclones sequenced on one strand. VDJ sequences were aligned with IgBLAST (49) software (IgBLAST, RRID : SCR_002873) against V, D, J genes in the IMGT (50) database (IMGT—the international ImMunoGeneTics information system, RRID : SCR_012780) and analysed for clonality (identical or related CDR3) and somatic mutations. The mixed *C57BL/6* and *129* background of the C*γ*1-Cre allele (44) was taken into account.

### Ig Isotype Quantification by Tandem Mass Spectrometry

Excised gel pieces were subjected to tryptic in-gel digest (51) followed by purification on C18 stage-tips (52). Samples were measured on a Q Exactive HF-x orbitrap mass spectrometer (ThermoFisher Scientific) connected to an EASY-nLC system (ThermoFisher Scientific). HPLC-separation occurred on an in‐house prepared nano‐LC column (0.074 × 250 mm, 3 μm Reprosil C18, Dr. Maisch GmbH) using a flow rate of 250 nl/min on a 45 min gradient with an acetonitrile concentration ramp from 4.7 to 46.5% (v/v) in 0.1% (v/v) formic acid. MS acquisition was performed at a resolution of 60,000 in the scan range from 350 to 1,800 m/z. MS2 scans were carried out at a resolution of 15,000 with the isolation window of 1.3 m/z and a maximum injection time of 100 ms. Dynamic exclusion was set to 20 s and the normalized collision energy was specified to 26.

For analysis, the MaxQuant software package (RRID : SCR_014485) version 1.6.3.4 was used (53, 54). An FDR of 0.01 was applied for peptides and proteins, and the andromeda search was performed using Uniprot (Universal Protein Resource, RRID : SCR_002380) (mouse database release July 2018, including isoforms). For protein identification a minimum of one unique peptide was required. Further analysis was done using R (R Project for Statistical Computing, RRID : SCR_001905). Proteins of non-mouse origin were considered contaminants and filtered out. All protein groups belonging to one immunoglobulin isotype were collapsed into one group by summing their individual intensities and were compared against the total intensity per sample.

### Statistical Analysis

Prism software (GraphPad Prism, RRID : SCR_002798) version 7 was used for pair-wise comparisons between mutant and control samples using non-parametric, unpaired, two-tailed Mann-Whitney U tests. Asterisks indicate statistical significance for p-values ≤0.05 (single), ≤0.01 (double), ≤0.001 (triple), ≤0.0001 (quadruple). Data are represented as individual points or means (bar graphs or horizontal lines) and error bars represent SD.

## Results

### Myd88^L252P^ Leads to NF-κB Activation and Short-Term Proliferation of Primary B Cells *Ex Vivo*

**Figure 1:**
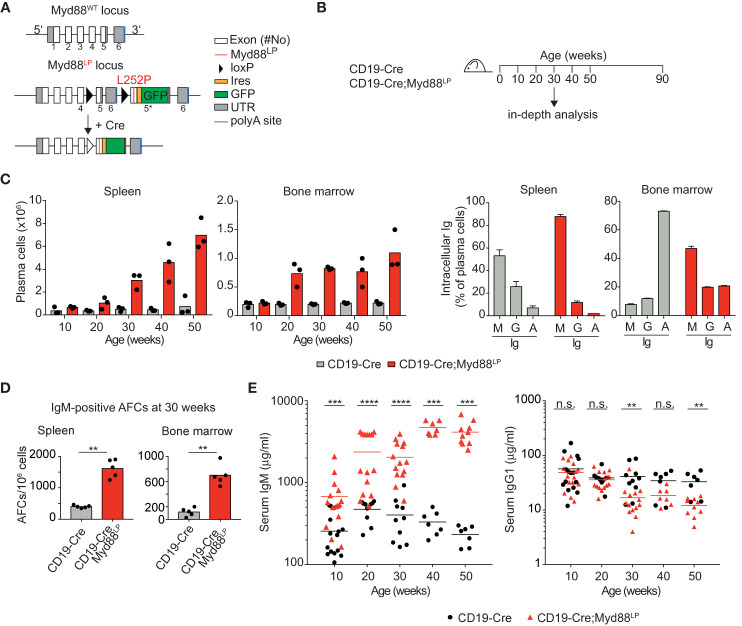
B-cell-specific Myd88^L252P^ expression causes increased IgM plasma cell and serum IgM levels. **(A)** Gene targeting strategy: Myd88^L252P^-IRES-GFP was targeted into the endogenous *Myd88* locus by homologous recombination. The wildtype exons 5 and 6 were flanked by loxP sites that can be recombined by Cre recombinase, leading to expression of the mutant version. **(B)** Outline of the experiments shown in C–E and **Table 1**. Mice of the indicated genotypes were observed for 90 weeks. **(C)** FACS analysis of spleen and bone marrow. Left: TACI^+^CD138^+^ plasma cell numbers increase over time. Right panels: Plasma cells expressed mostly IgM (30 weeks of age). **(D)** ELISPOT analysis in spleen and bone marrow at 30 weeks of age. IgM secreting antibody forming cells (AFCs) were elevated. **(E)** Serum immunoglobulin levels measured by ELISA. IgM titers increased over time while IgG1 titers decreased slightly. Results are representative of three independent experiments. **(C–E)** Each symbol represents one mouse. **p ≤ 0.01, ***p ≤ 0.001, ****p ≤ 0.0001, n.s. = not significant. (See also **Supplementary Figures 1–4**).

In order to investigate and track the consequences of the human MYD88^L265P^ mutation in mouse B cells, we generated a conditional Myd88 allele which expresses the mutation at the orthologous position L252P (as well as GFP) upon Cre-mediated recombination from the endogenous mouse Myd88 locus (**Figure 1A** and **Supplementary Figures 1A–D**). Endogenous Myd88^L252P^ expression induced a transient expansion of transgenic B cells in the absence or presence of added mitogens (**Supplementary Figure 1E**) consistent with the effect of retroviral overexpression of Myd88^L252P^ in mouse B cells *ex vivo* as previously reported (55). Myd88^L252P^ caused this effect at least partially by enhancing proliferation (**Supplementary Figure 1F**). As shown previously, these effects are likely due to Myd88^L252P^ activated NF-κB signaling (16–19, 55), concomitant with increased NF-κB negative regulatory feedback — through A20 (TNFAIP3) (55) and NF-κB p65 phosphorylation (56).

### B-Cell-Specific Myd88^L252P^ Expression *In Vivo* Leads to an Increase in IgM^+^ Plasma Cells and Serum IgM

In order to address whether B cell-specific expression of Myd88^L252P^ influences B cell development or homeostasis, we used the CD19-Cre allele (46) which is expressed from an early B cell stage on, and monitored mice until 90 weeks of age (**Figure 1B**). In this and all following experiments, mice heterozygous for the Cre and mutant Myd88 alleles were used, designated Cre;Myd88^L252P^. B cell development in the bone marrow appeared unchanged (**Supplementary Figure 2**), as indicated by the fractions of precursor, immature and mature B cells over time, absence of selection of AA4.1-positive Myd88^L252P^-expressing B lineage cells over YFP reporter expressing control cells and normal bone marrow histology. Two of thirteen mice developed a B cell lymphoma (at 70 and 74 weeks of age; **Table 1** and **Supplementary Figure 3A**). However, only one of these tumors expressed the Myd88^L252P^ reporter, indicating that these tumors arose spontaneously due to the *C57BL/6* genetic background (57).

**Table 1:** Myd88^L252P^ does not promote B lymphomagenesis.

Genotype	Number of animals	Age (weeks)	Phenotype at endpoint (90 weeks)
CD19-Cre	1	74	T cell tumor (TCRβ**^+^**)
	10	90	Healthy, end of experiment
CD19-Cre; Myd88^L252P^	1	70	T cell tumor (TCRβ**^+^**GFP**^-^**)
	1	74	GC B cell tumor (reporter-positive) (B220^+^CD19^+^CD38^low^FAS^high^GFP**^+^**)
	1	78	GC B cell tumor (reporter-negative) (B220^+^CD19^+^CD38^low^FAS^high^GFP**^-^**)
	1	90	T cell tumor (TCRβ**^+^**GFP**^-^**)
	9	90	Healthy, end of experiment

CD19-Cre and CD19-Cre;Myd88^L252P^ mice were observed for 90 weeks and monitored for the appearance of tumors. Tumors were analyzed and characterized by flow cytometry. Tumor incidence appeared comparable to control animals and likely was due to the genetic C57BL/6 background (57).

Starting at 30 weeks of age CD19-Cre;Myd88^L252P^ animals developed a mildly enlarged spleen with more than 95% of splenic B cells expressing the GFP reporter (**Supplementary Figures 3B, C**). While the percentage of follicular and marginal zone B cells appeared unchanged, germinal center (CG) B cells increased in frequency and number over time (**Supplementary Figures 3D–F**).

The most prominent phenotype in CD19-Cre;Myd88^L252P^ mice was an enlarged plasma cell compartment in the spleen, and to a lesser extent, in the bone marrow: Both the frequency and the absolute numbers of the TACI^+^CD138^+^ plasma cells from 50 weeks old CD19-Cre;Myd88^L252P^ mice were increased compared to CD19‑Cre control mice (**Figure 1C** and **Supplementary Figure 4A**). The majority of these expanded plasma cells expressed the Myd88^L252P^ reporter GFP, indicating that the plasma cell expansion was driven by the Myd88^L252P^ mutation (**Supplementary Figure 3C**). Strikingly, the majority of the expanded plasma cells also expressed and secreted IgM (**Figures 1C, D**; **Supplementary Figure 4B**). Correspondingly, serum IgM titers increased as early as ten weeks after birth and continued to increase over time up to twenty-fold, while other Ig isotypes were unchanged or slightly decreased (**Figure 1E**).

Taken together, our results suggest that the Myd88^L252P^ mutation causes elevated serum IgM levels and confers a subtle survival or growth advantage on IgM-expressing B cells that encompass a spectrum of differentiation states, including GC B cells and plasma cells.

### Ig Class Switching Is Unchanged in Myd88^L252P^-Expressing B Cells

**Figure 2:**
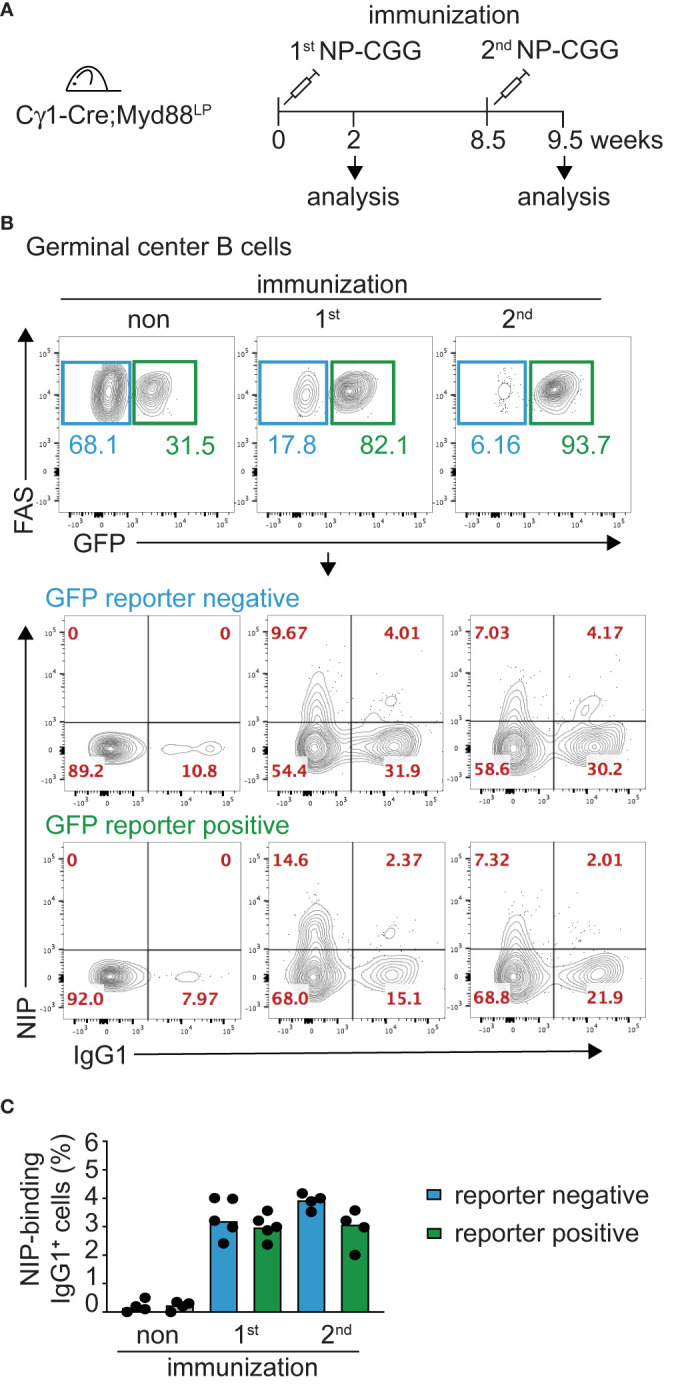
Myd88^L252P^-expressing B cells switch isotype normally. The Myd88^L252P^ allele was crossed into the B-cell-specific C*γ*1-Cre strain which activates Cre-expression in mature B cells upon germline transcription of the IgH Cγ1 switch region. **(A)** Cγ1-Cre;Myd88^L252P^ and littermate control animals were immunized with hapten carrier conjugate (NP-CGG) and analyzed at the indicated time points. **(B)** Representative flow cytometry plots of germinal center (GC) B cells (B220^+^CD19^+^CD38^low^FAS^high^). Upper panels: Myd88^L252P^ GFP-reporter-positive cells increased upon primary and secondary immunization. Lower panels: Antigen-specific, reporter-negative and -positive GC cells switch to IgG1 to similar extents during primary and secondary immune responses. **(C)** Percentage of IgG1-positive, antigen-positive GC cells. Each dot represents one mouse (n ≥ 4). (See also **Supplementary Figure 5**).

It has remained unclear whether the malignant B cells in WM are unable to switch Ig isotype from IgM to another class or whether switched WM cells might disappear over time *in vivo* (7, 8, 58–61). In order to gain insight into whether the Myd88^L252P^ mutation inhibits class switching, we crossed the Myd88^L252P^ mice with C*γ*1-Cre mice which express Cre in early GCs at a mature, activated B cell stage just prior to class switching (44). C*γ*1-Cre;Myd88^L252P^ animals were immunized with hapten-carrier conjugate as shown in **Figure 2A**. Antigen-specific GFP-reporter-positive and negative B cells did not differ in their ability to switch to IgG1 *in vivo*, neither after primary nor secondary immunization (**Figures 2B, C**). Supporting this result, *ex vivo* B cells transduced with TAT-Cre recombinase showed comparable Ig class switching efficiency in cell culture, irrespective of Myd88^L252P^ expression (**Supplementary Figure 5A**). We also assessed class switch in CD19-Cre mice (in which >95% of B cells are GFP-reporter-positive), and could not detect any change in the frequency of switched cells in either the spleen (IgG1), mesenteric lymph nodes (IgG1) or Peyer’s Patches (IgA) (**Supplementary Figure 5B**).

Collectively, these results indicate that the Myd88^L252P^ mutation does not interfere with Ig class switching. They rather suggest that the mutation specifically impacts the fitness of B cells expressing an IgM B cell receptor (BCR).

### Myd88^L252P^ Causes Prolonged Persistence of IgM^+^ Antigen-Specific Plasma Cells and Serum IgM

**Figure 3:**
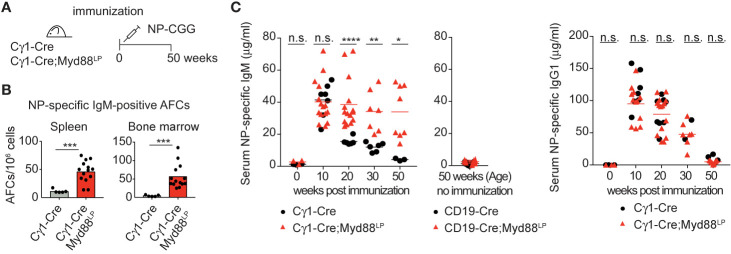
Myd88^L252P^ causes persistence of antigen-specific IgM plasma cells and serum IgM. **(A)** Cγ1-Cre;Myd88^L252P^ and littermate control animals were immunized with hapten carrier conjugate (NP-CGG) and analyzed at the indicated time points. **(B)** ELISPOT analysis in spleen and bone marrow at 50 weeks of age. Antigen-specific IgM secreting antibody forming cells (AFCs) were elevated. **(C)** Serum immunoglobulin levels measured by ELISA. Antigen-specific IgM titers remained elevated while IgG1 titers declined normally. Results are representative of three (NP-specific IgM) or two (NP-specific IgG1) independent experiments. **(B, C)** Each symbol represents one mouse (n ≥ 3). *p ≤ 0.05, **p ≤ 0.01, ***p ≤ 0.001, ****p ≤ 0.0001, n.s. = not significant. (See also **Supplementary Figure 6**).

In order to test directly whether IgM-expressing Myd88^L252P^-mutated B cells can persist for prolonged times *in vivo*, we followed reporter-positive antigen-specific B cells in C*γ*1-Cre;Myd88^L252P^ animals until 50 weeks after primary immunization with hapten-carrier conjugate NP-CGG (**Figure 3A**). As shown in **Figure 3B**, hapten-specific IgM-producing cells in spleen and bone marrow remained elevated up to 50 weeks after immunization. Consistent with this finding, NP-specific serum IgM titers remained elevated, while the NP-specific IgG1 titers decreased as in the controls (**Figure 3C**).

The same mice also showed an overall increase in the number of plasma cells (>80% reporter-positive) and elevated total serum IgM, similar to the CD19-Cre;Myd88^L252P^ mice described above, albeit to a lesser extent (**Supplementary Figures 6A, B**). BrdU-labeling over 16 h revealed an increased number of labeled splenic GC B cells and plasma cells compared to controls (**Supplementary Figure 6C**). Histology of the spleen suggested that this proliferation occurred mostly in plasma cell precursors, since CD138-positive plasma cells showed little active proliferation and were mostly Ki67-negative (**Supplementary Figure 6D**). Reminiscent of malignant Waldenström B cells, Myd88^L252P^ reporter-positive, IgM^+^ plasma cells carried increased numbers of somatic mutations compared to IgM^+^ plasma cells from controls (**Supplementary Figures 4C, 6E**).

Our results thus suggest that the Myd88^L252P^ mutation confers a survival and proliferation advantage to IgM-expressing B cells and plasma cell progenitors. Taking into account the capacity of these cells to switch isotype normally, these findings imply that surface IgM expression is required for the observed cellular expansion.

### Myd88^L252P^ Expression in a Small Number of B Cells Leads to Serum IgM Paraproteins (M-Spikes)

**Figure 4:**
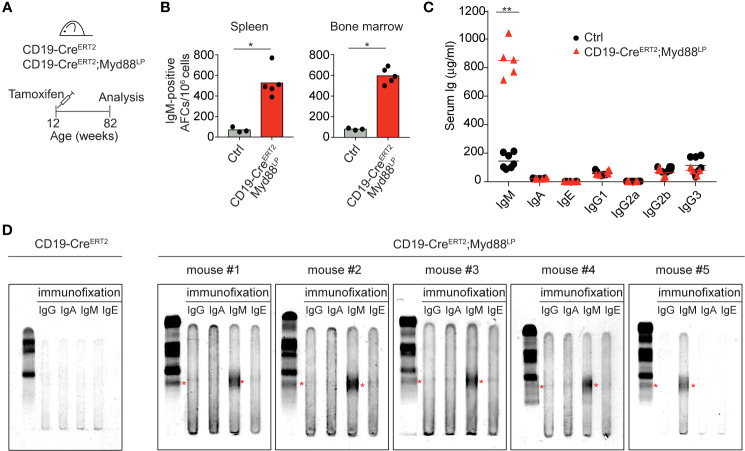
Myd88^L252P^-expression in a small number of B cells leads to serum IgM paraproteins (M-spikes). **(A)** CD19-Cre^ERT2^;Myd88^L252P^ animals were fed one time with tamoxifen and analyzed after 70 weeks. **(B)** ELISPOT analysis in spleen and bone marrow. IgM-secreting, antibody-forming cells are five to six times elevated in CD19-Cre^ERT2^;Myd88^L252P^ mice. **(C)** ELISA measurement of immunoglobulin serum titers. Only IgM was elevated in CD19-Cre^ERT2^;Myd88^L252P^ mice. **(B, C)** Each symbol represents one mouse (n ≥ 3). *p ≤ 0.05, **p ≤ 0.01. **(D)** Serum protein electrophoresis and immunofixation of serum from five CD19-Cre^ERT2^;Myd88^L252P^ mice. Each mouse showed a paraprotein band within the *γ*-globulin fraction (red stars) that was positive for IgM. Mouse #4 had an additional paraprotein band that was negative for IgM. (See also **Supplementary Figures 7** and **8**).

In WM patients, the MYD88^L265P^ mutation presumably arises as a rare event in a tumor progenitor cell. Therefore, to mimic the disease etiology more closely, we restricted mouse Myd88^L252P^ expression to a small fraction of B cells by a tamoxifen-inducible Cre allele (CD19-Cre^ERT2^) (47) which induces Cre-mediated recombination in only a few percent of B cells (**Figure 4**, **Supplementary Figure 7**). Ten days after a single dose of tamoxifen expression of Myd88^L252P^ led to a 15-fold increase in the reporter-positive plasma cell population in the spleen, an effect not observed in tamoxifen-treated YFP reporter control mice (**Supplementary Figure 7**). Importantly, 70 weeks after a single tamoxifen injection IgM-secreting plasma cells still persisted in spleen and bone marrow (**Figures 4A, B**). Correspondingly, serum IgM levels were also increased in the mutant animals (**Figure 4C**), all of which displayed discrete paraprotein bands in the *γ*-globulin zone upon serum protein electrophoresis. Such paraproteins are indicative of clonally restricted plasma cell expansions and occur in IgM MGUS, the precursor condition of WM (**Figure 4D** and **Supplementary Figure 8A**). Immunofixation confirmed that five out of five mice had developed a paraprotein of IgM isotype (**Figure 4D**).

IgM paraprotein bands occasionally also appeared in C*γ*1-Cre;Myd88^L252P^ mice, whereas we never observed paraprotein bands in sera of CD19-Cre;Myd88^L252P^ mice (**Supplementary Figure 8B**). Both in aged C*γ*1-Cre;Myd88^L252P^ and CD19-Cre^ERT2^;Myd88^L252P^ mice 70 weeks after tamoxifen injection, Myd88^L252P^-reporter-positive cells—while detectable only in low in numbers—consisted of B220**^+^** B cells and varying proportions of differentiated, mostly IgM-positive plasma cells (B220^low^TACI**^+^**CD138**^+^**) (**Supplementary Figures 8C, D**).

Thus, our data show that chronic activation of Myd88 in a small fraction of B cells can lead to the development of IgM M-spikes in the serum of aged, but otherwise healthy mice. They suggest a causal link between the Myd88^L252P^ mutation and IgM MGUS, the premalignant condition that precedes WM (23–26).

### Myd88^L252P^ Expression in a Small Number of B Cells Leads to Clonal Expansions of Plasma Cells

**Figure 5:**
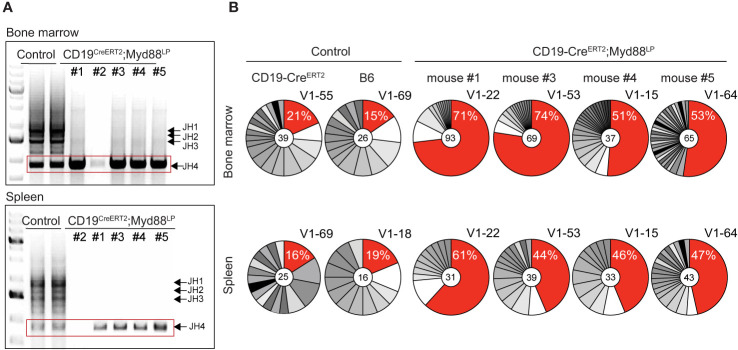
CDR3 analysis of rearranged VDJ genes shows expansion of clonally related plasma cells in aged Cre^ERT2^;Myd88^L252P^ mice. Genomic DNA was purified from GFP-reporter-positive TACI^+^CD138^+^ plasma cells isolated from bone marrow and spleen of CD19-Cre^ERT2^;Myd88^L252P^ mice 70 weeks after tamoxifen induction. **(A)** PCR amplification of rearranged J558 family V genes from bone marrow (upper panel) and spleen (lower panel). Bands corresponding to all four JH segments appeared in the controls, while in Cre^ERT2^;Myd88^L252P^ mice only J_H_4 rearrangements could be detected. (No rearrangements were detected in mouse #2). **(B)** J_H_4 bands (red rectangle shown in **A**) were cloned and sequenced. Clonal analysis based on CDR3 sequence revealed that in each mouse the same clones were most frequently detected in the bone marrow (upper panel) and spleen (lower panel). For the most frequently detected clonotype (red sector in pie chart), the % of sequences detected and the V_H_ J558 family member is given. V_H_ genes and VDJ rearrangements of the most frequent clonotypes were shared in bone marrow and spleen of each CD19‑Cre^ERT2^;Myd88^L252P^ mouse, while they differed in bone marrow and spleen of controls and between the controls. Each pie chart represents one mouse. (See also **Supplementary Figures 9** and **10**).

In order to determine the extent of clonal expansions in the plasma cell compartment in the five aged CD19-Cre^ERT2^;Myd88^L252P^ mice, we analyzed rearranged V_H_-region sequences in sorted plasma cells isolated from bone marrow and spleen. As read-out we examined the J558 family V genes which constitute about half of the expressed V_H_ gene repertoire in C57BL/6 mice (62–64). Amplification with a primer in the downstream J_H_ intron produced bands for all four J_H_ rearrangements in controls. By contrast, for four out of five CD19-Cre^ERT2^;Myd88^L252P^ mice, we only detected a single PCR band with J_H_4 being used in each case (**Figure 5A**). Since only a limiting amount of sorted plasma cells was available for this analysis, we cannot exclude that the J_H_4 bias may stem from preferential amplification of short VDJ rearrangements. (For the fifth mouse, we failed to obtain a PCR product.)

Subcloning and sequencing revealed that each of the four mice carried a different predominant J_H_4 rearrangement involving a J558 family member and that this predominant clonotype was overrepresented in plasma cells from both, bone marrow and spleen (**Figure 5B**). Plasma cells from age-matched control and CD19-Cre;Myd88^L252P^ and C*γ*1-Cre;Myd88^L252P^ mice also exhibited predominant clonotypes, but at a much lower frequency (12–25 versus 44–74% in mutants) and different ones in bone marrow and spleen (**Figure 5B** and **Supplementary Figure 9A**).

In striking contrast to the controls, the majority of plasma cells from CD19-Cre^ERT2^;Myd88^L252P^ mice expressed IgM (ranging from 68 and 88% in individual mice; **Supplementary Figure 9B**). Still, the overall extent of somatic mutation in GFP^+^ bone marrow-derived plasma cells from CD19-Cre^ERT2^;Myd88^L252P^ mice was comparable to control plasma cells which predominantly expressed IgG (**Supplementary Figure 9B**). In three mice the most frequently detected VDJ genes showed a moderate number of somatic mutations of up to 5, 13, or 15, respectively, which allowed the reconstruction of genealogical trees on the basis of intraclonal variation (**Supplementary Figure 9C**). In one mouse (#4) the most frequent VDJ gene was unmutated.

In order to find out whether the IgM M-spikes observed in these four mice contained the same clonal VDJ rearrangements that were predominantly detected in spleen and bone marrow of the individual mice, we analyzed protein bands corresponding to the individual M-spikes by tandem mass spectrometry (**Supplementary Figure 10**). Proteomics confirmed in all cases that the predominant isotype in the M-spike was IgM, but did not reveal clonotypic peptides corresponding to the VDJ regions that were most frequently detected by sequencing.

Notwithstanding the absence of a clear molecular link between the M-spikes and the most frequently detected plasma cell clones in bone marrow and spleen of the four CD19-Cre^ERT2^;Myd88^L252P^ mice, our clonal analysis suggests that—in a genetic scenario where introduction of Myd88^L252P^ mutation into CD19^+^ B cells is a rare event—Myd88^L252P^ mutation confers a survival and growth advantage to rare cells that over time produce clonal expansions of IgM-positive plasma cell progenitors (**Supplementary Figure 9C**).

## Discussion

IgM and non-IgM MGUS are different clinical entities that are both thought to arise from B cells at late stages of differentiation (23, 25, 65). While non-IgM MGUS mostly evolves to multiple myeloma (25, 66–68), IgM MGUS has been increasingly recognized as the premalignant precursor state for WM (23–26, 65). However, to date it has remained challenging to clinically or molecularly distinguish WM from smoldering WM and IgM MGUS (23, 69–71). Premalignant IgM MGUS and malignant WM cells were found to be phenotypically similar to each other (70).

The MYD88^L265P^ mutation is absent in multiple myeloma patients (27), but highly prevalent in both, WM and IgM-MGUS patients (27–33). It therefore may represent an early, unifying genetic event in WM pathogenesis. Here, we provide evidence that B-cell-specific expression of the mouse homolog of the human MYD88^L265P^ mutation (Myd88^L252P^) is sufficient to cause a phenotype that resembles IgM MGUS. We thus establish a causal link between the Myd88^L265P^ mutation and the development of a phenotype resembling the WM precursor condition and shed new light on the etiology of WM.

Based on three different genetic scenarios, our results indicate that chronic activation of aberrant Myd88 signaling—by conditional mutagenesis of the endogenous Myd88 locus—confers a survival and low-grade proliferative advantage on IgM-expressing B cells. This advantage can manifest in different ways, depending on the number cells targeted by the mutation and the time window for progression: In a first scenario, activation of the mutation by CD19-Cre in early B cells caused a polyclonal, low-grade lymphoproliferative disease accompanied by polyclonal plasma cell expansion and progressively increasing serum IgM titers (up to 20-fold). In a second scenario, activation of the mutation at the initiation of the GC stage by C*γ*1-Cre caused a similar, albeit weaker, phenotype, consistent with a lower number of mutated B cells. In a third scenario, a time-restricted activation of Myd88^L252P^ by CD19-Cre^ERT2^ in a small fraction of B cells led to clonal expansions of IgM-expressing plasma cells and the appearance of IgM M-spikes in the serum.

The latter scenario most closely mimics the *in vivo* situation in human patients, where Myd88 mutation presumably occurs as a rare event in a tumor progenitor B cell. It appears that IgM expressing Myd88^L252P^ mutated B cells can gain a competitive advantage over normal B cells over time resulting in an outgrowth of clonally related, mutated cells and IgM M-spikes in the blood when the mutation is restricted to few or single progenitor B cells. Polyclonal activation of Myd88^L252P^ (as in the first and second scenario) may mask this effect, and indeed resulted in overall strongly elevated IgM levels (**Figure 1E** and **Supplementary Figure 6B**) (19). In support of this interpretation, we never detected IgM M-spikes when the mutation was activated by the CD19-Cre allele (causing recombination in most B cells) and only occasionally when the mutation was activated by the C*γ*1-Cre allele (which is active in fewer B cells) (44).

The presence of IgM M-spikes in the blood of aged CD19-Cre^ERT2^;Myd88^L252P^ mice was accompanied by clonal expansions in the plasma cell compartment with the most frequently detected clonotype being identical in spleen and bone marrow. Clonally related plasma cells mostly carried somatically mutated VDJ regions, reminiscent of a molecular WM cell phenotype (6–11). We also observed intraclonal diversity with respect to somatic mutations (**Supplementary Figures 9B, C**), suggesting that the Myd88^L252P^ mutation drives IgM MGUS progenitors already at the GC stage, consistent with our finding that GC B cells rather than plasma cells are actively proliferating (**Supplementary Figures 6C, D**). However, our results do not exclude that the pro-proliferative activity of the Myd88^L252P^ mutation extends into later stages of B cell differentiation.

Our attempts to find a direct molecular link by proteomics between the IgM M-spikes and the most frequently detected clones were unsuccessful. This may be due to the low amount of starting material combined with the complexity of serum samples, the presence of multiple clonotypes in the isolated M-spike, the locally restricted area of the bone marrow biopsy (femur), or a combination of these factors. It is also possible that in CD19-Cre^ERT2^;Myd88^L252P^ mice the most frequently detected plasma cell clones form part of an early, dynamic clonal landscape in which several competing Myd88^L252P^ B cell clones are still present until secondary mutations help to establish dominance and long-term persistence of a single major clone.

Our study is in line with the prevailing view that the development of WM requires additional mutations besides MYD88^L265P^ (19, 20, 36, 39–41, 55). The observed B cell phenotypes are consistent with earlier work that assessed the effect of retroviral overexpression of mouse Myd88^L252P^ in B cells *ex vivo* (55) or B-cell-specific transgenic overexpression of human MYD88^L265P^ *in vivo* (20). Both approaches showed that the Myd88 mutation by itself is not sufficient to immortalize or neoplastically transform B cells. This appears plausible, because activation of pro-survival signaling by NF‑κB entails negative feedback that limits B cell expansion (55). The need to remove negative feedback loops may explain the frequent occurrence of mutations that affect negative regulators of NF-κB in human WM or ABC-DLBCL patients (36, 37, 72–75). Our results (in genetic scenarios one and two) are also in line with the observation that human MYD88^L265P^ promotes in the mouse the development of a polyclonal, low-grade B cell lymphoproliferative disorder of lymphoplasmacytic appearance with increased serum IgM (20).

However, different from earlier studies, continuous activation of an endogenous Myd88^L252P^ mutation by CD19-Cre in our mouse cohort did not cause fulminant lymphoproliferative disease (19) or an increased transformation to B lymphoma or increased mortality (**Table 1**; **Supplementary Figure 3A**) (19, 20). Rather than owing to differences in the human and mouse Myd88 proteins, as proposed recently by Sewastianik et al. (20), these discrepancies may be caused by different external cues (such as TLR signaling induced by different microbial or mouse housing environments) (19) or the molecular effects of strong transgene overexpression (20), or both.

Our results suggest that IgM expression is specifically required for the pro-survival effect of the Myd88^L252P^ mutation, since mutated B cells showed normal Ig isotype switching in a wide range of experimental conditions, but only IgM-expressing, antigen-specific B cells were able to persist after immunization. In line with these results, Young et al. (76) proposed that cell surface IgM acts as an “initiator oncogene” for B cell lymphomas, with the IgM-BCR potently promoting B cell proliferation and IgG-BCRs preferentially promoting B-cell differentiation programs. In this view, the IgM-BCR acts “as an oncogene that initiates proto-malignant expansion of normal B cells”, while the extended survival of pre-malignant cells would require additional cooperating oncogenic events.

One such event may be the MYD88^L265P^ mutation which transforms normal IgM-expressing proliferating B cells into premalignant cells that show prolonged survival and plasmacytic differentiation. This effect may be driven by external triggers through TLR signaling and be dependent on BCR surface expression. Enforced overexpression of Myd88^L525P^ in B cells under the control of a strong constitutive viral promoter (77) may overcome such a dependency on external triggers (13) and manifest directly in a Waldenstöm-like B cell lymphoma (77). In ABC-DLBCL cells, and in at least one WM-derived cell line, the MYD88^L265P^ mutation promotes the formation of an oncogenic signaling complex comprising Myd88, TLR9 and an IgM-BCR (My-T-BCR super complex) which enforces cooperative survival signaling through the BCR and TLR (16, 17, 76, 78, 79). It will be interesting to determine in this context whether a My-T-BCR super complex already forms in Myd88-mutated B or plasma cells expressing physiological levels of mutated Myd88, or whether super complex formation requires either additional oncogenic mutations or increased expression of mutated Myd88, or both.

## Data Availability Statement

All nucleotide datasets generated for this study can be found in the **Supplementary Material** (**Supplementary Tables 2** and **4**). The mass spectrometry proteomics data can be found in **Supplementary Material** (**Supplementary Table 3**) and raw data were deposited to the ProteomeXchange Consortium via the PRIDE (80) partner repository with the dataset identifier PXD017292 (Proteomics Identifications (PRIDE), RRID:SCR_003411).

## Ethics Statement

The animal study was reviewed and approved by Landesamt für Gesundheit und Soziales Berlin (LaGeSo# G0263/15). 

## Author Contributions

KS, US, CK, and KR designed research. US performed Myd88^L252P^ gene targeting. KS performed all experiments except Myd88^L252P^ gene targeting, prepared all figures, and a first manuscript draft. OP and PM carried out the proteomic analyses. KS, CK, KR, OP, and PM analyzed data. TS, RG, and WW provided expertise. CK wrote and KR edited the final manuscript with critical contributions from KS, TS, RG, and WW. All authors contributed to the article and approved the submitted version.

## Funding

This work was supported by the European Research Council, Advanced Grant 268921 (to KR), the Helmholtz Association, Immunology & Inflammation ZT-0027 (to KR), the Deutsche Krebshilfe, Grant 70112800 (jointly to KR and M. Janz, Max Delbrück Center for Molecular Medicine in the Helmholtzgemeinschaft, Berlin, Germany), the Berlin School of Integrative Oncology and Charité Universitätsmedizin Berlin, Promotionsstipendium I (to KS) and the library of the Max Delbrück Center for Molecular Medicine in the Helmholtz Association, Open Access Fonds (to KR).

## Conflict of Interest

The authors declare that the research was conducted in the absence of any commercial or financial relationships that could be construed as a potential conflict of interest.
