# Supplementary material for: B-Cell-Specific Myd88 L252P Expression Causes a Premalignant Gammopathy Resembling IgM MGUS
Source: Front Immunol. 2020 Dec 1;11:602868. doi: 10.3389/fimmu.2020.602868 (PMC7747680; doi:10.3389/fimmu.2020.602868)

https://www.frontiersin.org/articles/10.3389/fimmu.2020.602868/full#supplementary-material

Supplementary Material

The Supplementary Material includes four Supplementary Tables with Legends (three separate Excel files) and ten Supplementary Figures with Legends.

SUPPLEMENTARY TABLES

Supplementary Table 1 | Reagents, probes and other resources. Reagents, probes and other resources for different experimental or other procedures are listed with source and identifier.

**REAGENT or RESOURCE**	
**Flow cytometry antibodies**	
Specificity	
CD93 (AA4.1) - PE/Cy7 conjugated	BioLegend Cat# 136506; RRID: AB_2044012
CD45R/B220 - Brilliant Violet 785™ conjugated	BioLegend Cat# 103245; RRID: AB_11218795
BrdU Flow Kit - APC conjugated	BD Biosciences Cat552598; RRID: AB_2861367
BrdU Monoclonal Antibody (BU20A) - FITC conjugated	eBioscience Cat# 11-5071-42; RRID: AB_11042627
CD117 (c-kit) - APC conjugated	BioLegend Cat# 135108; RRID: AB_2028407
CD138 (Syndecan-1) - Brilliant Violet 421™ conjugated	BioLegend Cat# 142508; RRID: AB_11203544
CD19 - Brilliant Violet 605™ conjugated	BioLegend Cat# 115540; RRID: AB_2563067
CD1d (CD1.1, Ly-38) - PE conjugated	BioLegend Cat# 123510; RRID: AB_1236538
CD21/CD35 (CR2/CR1) - Brilliant Violet 421™ conjugated	BioLegend Cat# 123421; RRID: AB_10965544
CD38 - Alexa Fluor 700 conjugated	eBioscience Cat# 56-0381-82; RRID: AB_657740
CD95 - PE-Cy™7 conjugated	BD Biosciences Cat# 557653
IgA (mA-6E1) - PE conjugated	eBioscience Cat# 12420482; RRID: AB_465917
IgD - PerCP/Cy5.5 conjugated	BioLegend Cat# 405710; RRID: AB_1575113
IgG - PE conjugated	BD Biosciences Cat# 550083
IgG2a - PE conjugated	BioLegend Cat# 407107; RRID: AB_10549974
IgG2b - PE conjugated	BioLegend Cat# 406707; RRID: AB_2563380
IgG3 - PE conjugated	SouthernBiotech Cat# 1191-09L; RRID: AB_2794697
IgM - Biotin	SouthernBiotech Cat# 1020-08; RRID: AB_2737411
TACI (CD267) - Alexa Fluor® 647 conjugated	BD Biosciences Cat# 558453

**Bacterial Strains and Cloning**	
Subcloning Efficiency DH5α Competent Cells	Invitrogen Cat# 18265017
One Shot®Top10	Invitrogen Cat# C404010
Zero Blunt TOPO PCR Cloning Kit for Sequencing	Invitrogen Cat# 450031
**ELISA and ELISPOT antibodies**	
**Detection reagents**	
Goat anti-mouse IgA-Biotin	SouthernBiotech Cat# 1040-08; RRID: AB_2794374
Goat anti-mouse IgA	SouthernBiotech Cat# 1040-01; RRID: AB_2314669
Goat anti-mouse IgE-Biotin	SouthernBiotech Cat# 1110-08; RRID: AB_2794605
Goat anti-mouse IgE	SouthernBiotech Cat# 1110-01; RRID: AB_2794601
Goat anti-mouse IgG_1-Biotin	SouthernBiotech Cat# 1071-08; RRID: AB_2794427
Goat anti-mouse IgG_{2a}-Biotin	SouthernBiotech Cat# 1080-08; RRID: AB_2794479
Goat anti-mouse IgG_{2a}	SouthernBiotech Cat# 1081-01; RRID: AB_2794492
Goat anti-mouse IgG_{2b}-Biotin	SouthernBiotech Cat# 1090-08; RRID: AB_2794523
Goat anti-mouse IgG_{2b}	SouthernBiotech Cat# 1091-01; RRID: AB_2794538
Goat anti-mouse IgG_3-Biotin	SouthernBiotech Cat# 1100-08; RRID: AB_2794575
Goat anti-mouse IgG_3	SouthernBiotech Cat# 1101-01; RRID: AB_2794586
Goat anti-mouse IgM	SouthernBiotech Cat# 1020-08; RRID: AB_2737411
**Standards**	
Mouse IgM	SouthernBiotech Cat# 0101-01; RRID: AB_2629437
Mouse IgG_1	SouthernBiotech Cat# 0102-01; RRID: AB_2793845
Mouse IgG2a	SouthernBiotech Cat# 0103-01; RRID: AB_2736850
Mouse IgG2b	SouthernBiotech Cat# 0104-01; RRID: AB_2793882
Mouse IgG3	SouthernBiotech Cat# 0105-01; RRID: AB_2793898
Mouse IgA	SouthernBiotech Cat# 0106-01; RRID: AB_2714214
Mouse IgE	SouthernBiotech Cat# 0114-01; RRID: AB_2793972
**Coating reagents**	
Goat anti-mouse Kappa	SouthernBiotech Cat# 1050-01; RRID: AB_2737431
Goat anti-mouse Lambda	SouthernBiotech Cat# 1060-01; RRID: AB_2794389
**Immunohistochemistry antibodies**	
Specificity	
CD11b – Brilliant Violet 421™ conjugated	BioLegend Cat# 101235; RRID: AB_10897942
CD138 – PE conjugated	BioLegend Cat# 142504; RRID: AB_10916119

CD38 – APC conjugated	BioLegend Cat# 102712; RRID: AB_312933
CD3e – FITC conjugated	eBioScience Cat# 100305; RRID: AB_312670
CD95 – PE conjugated	BD Biosciences Cat# 554258; RRID: AB_395330
IgM – APC conjugated	eBioScience Cat# 17579082; RRID: AB_469458
Monocytes and macrophages (clone MOMA-2) - Biotin	BMA Biomedicals Cat# T-2029, RRID:AB_1227438
TCRβ – FITC conjugated	eBioScience Cat# 465322; RRID: AB_465322
Ki67 – PE conjugated	BioLegend Cat# 652404; RRID: AB_2561525
Biotin - Brilliant Violet 421™ conjugated Streptavidin	BioLegend Cat# 405225
**Software, Algorithms and databases**	
Prism 7.0a Software for Statistical Analyses	GraphPad Prism, RRID:SCR_002798
FlowJo 10.3 Software for Cytometry Analysis	BD FlowJo, RRID:SCR_008520
IgBLAST Software Tool for Immunoglobulin Analysis	NCBI (NIH), IgBLAST, RRID:SCR_002873
ApE (A plasmid Editor) Software for DNA sequences	M. Wayne Davis, A plasmid Editor, RRID:SCR_014266
ImmunoSpot Software for Analyzing ELISPOT Assays	CTL Europe GmbH, RRID:SCR_011082
MaxQuant Software for Proteomics Analysis	MPG/MPI of Biochemistry, RRID:SCR_014485
R Project for Statistical Computing (R)	The R Foundation, RRID:SCR_001905
Standardized analysis of rearranged IG VDJ genes	IMGT/V-QUEST, RRID:SCR_010749
**Southern blot probes**	
5’Probe1 (this study)<br><br>CTAGACCCAAGGAAGATTTTTTCGTTAATTCACTTGGTTAATCTATTTAGGTGTCAATCTCCAATACTGGGTCTCCCTC<br>CACCCTCCACCCCCATCCCCGGGTTTAGTAGAATTCCTAGGGGAATGTGGGCACTAATGGCACTATCCCAAACAAAC<br>GTAGATCAACAGGCTTAAGACGAGTGGGGCACGTTACAGTTAGAACCTGCCTGCCGTTCAGCACCTTAATCGGTTCTT<br>CACACCAGCCCGGAACCCAAAATTACTTTGTTTTCTGAGAGAGCTTCTGTGACAGCCAACCCGGTTGGGGCTTAACTA<br>GGTGCCTCAACATTTTCTAGGTCCTCAGTTCTCGGTTTTGGGACCCGGGGCTGAGCACAGCAAATTTTGATGGGAGAC<br>AAGGGTCTTCTCGGGCTAGCTCAGCAGAGAAGCCATGGGCCGGGCTCCGCCTTCTACGCGGCTGCGCGCTGAGCTCA<br>AGGGGAGGGACTCCCTTGAGGCTGAGTTCATCCTAGGAAACCAGAAGTTTGGGAAACCTCCTTCCCCAATGTCTACA<br>AAAGGTTGTGCAAACTTGGAAGTTTCCAGTCTGCCTTTCAAGGAGGAT	
3’Neo (this study)<br><br>GGATCCGAACAAACGACCCAACACCCGTGCGTTTTATTCTGTCTTTTTATTGCCGATCCCCTCAGAAGAACTCGTCAA<br>GAAGGCGATAGAAGGCGATGCGCTGCGAATCGGGAGCGGCGATACCGTAAAGCACGAGGAAGCGGTCAGCCCATTC<br>GCCGCCAAGCTCTTCAGCAATATCACGGGTAGCCAACGCTATGTCCTGATAGCGGTCCGCCACACCCAGCCGGCCAC<br>AGTCGATGAATCCAGAAAAGCGGCCATTTTCCACCATGATATTCGGCAAGCAGGCATCGCCATGGGTCACGACGAGA<br>TCCTCGCCGTCGGGCATGCGCGCCTTGAGCCTGGCGAACAGTTCGGCTGGCGCGAGCCCCTGATGCTCTTCGTCCAGA<br>TCATCCTGATCGACAAGACCGGCTTCCATCCGAGTACGTGCTCGCTCGATGCGATGTTTCGCTTGGTGGTCGAATGGG<br>CAGGTAGCCGGATCAAGCGTATGCAGCCGCCGCATTGCATCAGCCATGATGGATACTTTCTCGGCAGGAGCAAGGTG<br>AGATGACAGGAGATCCTGCCCCGGCACTTCGCCCAATAGCAGCCAGTCCCTTCCCGCTTCAGTGACAACGTCGAGCA<br>CAGCTGCGCAAGGAACGCCCGTCGTGGCCAGCCACGATAGCCGCGCTGCCTCGTCCTGCAG	

Supplementary Table 2 (related to Supplementary Figure 4-6 and 9) | Identity of rearranged VH, D and JH4 sequences cloned from plasma cells of control and Myd88L252P mice. Genomic DNA was isolated from sorted splenic plasma cells ($TACI^{+}CD138^{+}$). Rearranged J558 V family genes were amplified from genomic DNA with primers specific for J558 V family genes and for the IgH intronic enhancer. A 0.9 kb band corresponding to J_H4 rearrangements was gel-purified, cloned into plasmid vector pCR4Blunt-TOPO (Invitrogen) and sequenced. VDJ sequences were aligned with IgBLAST against IMTG V, D or J genes and the J_H intron sequences compared to the germline. The table lists V_H genes and CDR3 sequences, as well as number of mutations in V_H genes and J_H introns, according to genotypes (lower right tabs for 3 different sheets): CD19-Cre control and CD19-Cre;$Myd88^{L252P}$ (data shown in **Supplemental Figures 4C and 9A**), Cγ1-Cre control and Cγ1-Cre;$Myd88^{L252P}$ (data shown in **Supplemental Figures 6E and 9A**), and CD19-CreERT2 control and CD19-Cre^{ERT2};$Myd88^{L252P}$ (data shown in **Figure 5B and Supplemental Figure 9B-C**). Most frequently detected clonotypes are highlighted in red.

See separate Excel file.

Supplementary Table 3 (related to Supplementary Figure 10) | Proteomics analysis of M-spike bands by tandem mass spectrometry. Combined intensities for each Ig subtype or protein group. Protein groups (after filtering out contaminants and non-relevant protein identities) and their assignment to protein classes. Intensities were not normalized. The mass spectrometry proteomics data were deposited to the ProteomeXchange Consortium via the PRIDE partner repository **(1)** with the dataset identifier PXD017292 (Proteomics Identifications (PRIDE), RRID:SCR_003411).

See separate Excel file.

Supplementary Table 4. Complete IgH VDJ and intron nucleotide sequences | Complete nucleotide sequences of all rearranged VDJ sequences sequenced in this study. Nucleotide sequences were compiled in FASTA format and analyzed with IMGT/V-QUEST (RRID:SCR_010749) version 3.5.19 (July 2020) and reference directory release 202031-2 (28 July 2020)). Result summeries were exported to an Excel file using IMGT/V-QUEST Default parameters.

See separate Excel file.

SUPPLEMENTARY FIGURE LEGENDS

Supplementary Figure 1 (related to Figure 1) | A new conditional Myd88^{L252P} allele induces short-term proliferation of B cells through activation of NF-κB. **(A)** Southern blots of genomic DNA from Myd88^{L252P} ES cell clone #5 and R26^{BFP} ES cells. Restriction enzymes (FspAI, Afl II) and hybridization probes (5′Probe1, 3′Neo) are indicated. Bands corresponding to the expected targeted integration into the Myd88 locus are indicated (Target). Probe sequences are given in supplemental Table 1. **(B-C)** Splenic B cells from *C57BL/6* control (Myd88$^{+/+}$), heterozygous (Myd88$^{LP/+}$), and homozygous (Myd88$^{LP/LP}$) Myd88^{L252P}-floxed mice were isolated and transduced with TAT-Cre recombinase *ex vivo* and cultured. Data are representative of 3 independent experiments or mean for triplicate cultures per group. **(B)** cDNA sequence of Myd88 mRNA isolated from reporter-positive B cells sorted four days post TAT-Cre transduction. Samples from heterozygous and homozygous mice show the expected T to C gain-of-function point mutation (Leu252Pro). **(C)** Reverse transcription, real-time quantitative PCR (triplicate measurements) normalized to β-actin mRNA. The expression level of Myd88 mRNA was not changed after mutation of the endogenous Myd88 locus and the GFP reporter was expressed as expected. **(D-F)** B cells isolated from heterozygous *R26^{YFP}*- or Myd88^{L252P}-floxed (Myd88^{LP}) animals were transduced with TAT-Cre. **(D)** Cells were analyzed 48 hours later for GFP-reporter expression. 68% of B cells were GFP-positive. **(E)** B cells were stimulated with lipopolysaccharide (LPS) or with anti-IgM antibodies and monitored for cell growth. Myd88^{L252P}-expressing B cells showed increased short-term proliferation compared to control B cells. Minor proliferative activity in the absence of added mitogens may be due to bacterial impurities in the TAT-Cre preparation. **(F)** Four days post TAT-Cre transduction B cells were labeled with BrdU for four hours. Consistent with increased short-term proliferation as shown in panel **E**, Myd88^{L252P}-mutated B cells showed higher levels of BrdU incorporation than control B cells.

Supplementary Figure 2 (related to Figure 1) | B cell development in the bone marrow of CD19-Cre;Myd88^{L252P} mice appears normal. **(A)** Representative flow cytometry plots at 30 weeks of age depicting the frequency of total B lineage cells (B220^{+} green), mature B cells (B220^{+}AA4.1^{-}; yellow) and AA4.1-positive B lineage cells (B220^{+}AA4.1^{+}; red). AA4.1-positive B lineage cells were further divided into immature B cells (B220^{+}AA4.1^{+}IgM^{+}IgD^{-}; blue), Pre B cells (B220^{+}AA4.1^{+}IgM^{-}IgD^{-}cKit^{+}; magenta) and Pro B cells (B220^{+}AA4.1^{+}IgM^{-}IgD^{-}cKit^{-};black). Cell fractions were comparable to control mice. **(B)** Percentage of total B lineage cells, mature B cells and AA4.1-positive B lineage cells over time in CD19-Cre (grey) and CD19-Cre;Myd88^{L252P} animals (red). Each dot represents one mouse (n=3). Cell fractions were comparable to control mice. **(C)** Percentage of reporter expression within the indicated B cell populations in 30-week-old CD19-Cre;R26YFP (grey) and CD19-Cre;Myd88^{L252P} animals (red). Each dot represents one mouse (n=3). Myd88^{L252P} GFP reporter expression was comparable to R26 YFP reporter controls, indicating an absence of selection of Myd88-mutated precursor B cells over non-mutated cells. **(D)** Histology (haematoxylin and eosin staining) of a representative bone marrow section (30 weeks of age). Scale bar: 100 µm. The bone marrow appeared intact and unchanged in the mutant mouse.

Supplementary Figure 3 (related to Figure 1) | B-cell-specific expression of Myd88^{L252P} causes an increase in germinal center B cells. **(A)** Percentage of symptom-free CD19-Cre;Myd88^{L252P} (red; n=13) and littermate control CD19-Cre mice (black; n=11). Mutant and control mice behaved similarly (no significant difference by Mantel Cox log rank or Gehan-Brelow-Wilcoxon test). See Table 1 for details. **(B)** Spleen weight (left) and total splenocytes (right) of CD19-Cre (grey) and CD19-Cre;Myd88^{L252P} (red) animals at the indicated time points. Each dot represents one mouse

(n=3). Spleen weight and total splenic cell number increased with age. **(C)** Percentage of reporter expression within the indicated splenic B cell populations in 30-week-old CD19-Cre;R26^{YFP} (grey) and CD19-Cre;Myd88^{L252P} (red) animals. Each dot represents one mouse (n=3). Reporter expression was high in all B cell types, indicating efficient Cre-induction in CD19-Cre mice. **(D)** Representative flow cytometry plots at 30 weeks depicting the frequency of splenic total B cells (B220^{+}CD19^{+}). Splenic B cells were further divided in follicular (CD21^{+}CD1d^{-}), marginal zone (CD21$^{\mathbf{high}}$Cd1d$^{\mathbf{high}}$) and germinal center B cells (FAS$^{\mathbf{high}}$CD38$^{\mathbf{low}}$). Germinal center B cells appeared increased while marginal zone B cells appeared normal. **(E)** Percentage over time at ten week-intervals of splenic germinal center B cells (B220^{+}CD19^{+}FAS$^{\mathbf{high}}$CD38$^{\mathbf{low}}$) in CD19-Cre (grey) and CD19-Cre;Myd88^{L252P} mice (red). Each dot represents one mouse (n=3). Germinal center B cells increased over time. **(F)** Immunohistochemistry (left) of representative spleen sections at 30 weeks stained for CD38 (green) and FAS (purple). Scale bar: 100 µm. Quantification (right) of germinal center B cell area. Each dot represents one germinal center. Six to eight germinal centers were analyzed per mouse (n=3). Area of germinal centers in CD19-Cre;Myd88^{L252P} mice was increased in size compared to control.

Supplementary Figure 4 (related to Figure 1) | B-cell-specific expression of Myd88^{L252P} leads to plasma cell expansion. **(A-B)** Immunohistochemistry of splenic sections from 30-week-old CD19-Cre;Myd88^{L252P} mice. Scale bars: 100 µm. **(A)** Left panel: Splenic sections stained for DNA (DAPI; blue) and plasma cells (CD138; yellow). Plasma cells were increased in numbers in CD19-Cre;Myd88^{L252P} mice compared to control. **(B)** Splenic sections stained for IgM (green), plasma cells (CD138; yellow), macrophages (MOMA-2; light blue) and T cells (CD3ε; red). Accumulations of IgM^{+}CD138^{+} plasma cells in the red pulp are indicated with dotted lines. **(C)** Extent of somatic hypermutation in the introns downstream of JH4 in sorted TACI^{+}CD138^{+} plasma cells of the indicated genotypes (in presence of Myd88^{LP} also GFP reporter-positive). J558 V family genes rearranged to JH4 were amplified from genomic DNA, cloned and sequenced. One mouse per genotype was analyzed. The number of sequences analyzed is indicated in the center of the pie chart. The number of mutations is color-coded ranging from white (no mutations) to black (more than 10 mutations). IgM-positive cells from the CD19-Cre;Myd88^{L252P} mouse carried a higher level of somatic hypermutation than IgM-positive cells from the CD19-Cre control mouse.

Supplementary Figure 5 (related to Figure 2) | Myd88^{L252P} does not affect immunoglobulin class-switch recombination *ex vivo* and *in vivo.* **(A)** Representative flow cytometry plots (left) and quantification (right) of splenic B cells isolated from *C57BL/6* control (grey) and Myd88^{L252P} animals (red). Cells were transduced with TAT-Cre and stimulated with LPS and IL-4. Switching to IgG1 was monitored four days after stimulation. Each dot represents one mouse (n=5). Mutant and control B cells *ex vivo* switched equally well to IgG1. **(B)** Flow cytometry plots (left) and quantification (right) of surface IgM, IgG1 or IgA staining of germinal center B cells (B220^{+}CD19^{+}FAS$^{\mathbf{high}}$CD38$^{\mathbf{low}}$) in spleen (upper panel), mesenteric lymph nodes (middle panel) and Peyer's Patches (lower panel) from 30 week-old CD19-Cre (grey) and CD19-Cre;Myd88^{L252P} mice (red). Each dot represents one mouse (n=3). For CD19-Cre;Myd88^{L252P} mice, only reporter-positive cells are shown. Mutant and control B cells *in vivo* switched equally well to IgG1 or IgA.

Supplementary Figure 6 (related to Figure 3) | Induction of Myd88^{L252P}-expression in mature B cells causes increased IgM plasma cell formation, proliferation and extent of somatic hypermutation. The Myd88^{L252P} allele was bred into the Cγ1-Cre strain that activates Cre expression in mature B cells upon germ line transcription of the IgH Cγ1 switch region. Mice were immunized with NP-

CGG and analyzed 50 weeks after immunization (as outlined in **Figure 3A**). **(A)** Representative flow cytometry plots (left) and quantification (right) of reporter-positive splenic plasma cells ($TACI^+CD138^+$). Each dot represents one mouse (Cγ1-Cre control: n=5; Cγ1-Cre;$Myd88^{L252P}$: n= 14). Mutant plasma cells were slightly elevated in number compared to control. **(B)** Left panel: ELISPOT analysis of IgM-positive antibody-forming cells. Averages of duplicate measurements per mouse are shown as black dots (n≥5). Right panel: ELISA measurement of IgM and IgG1 serum titers in Cγ1-Cre (black) and Cγ1-Cre;$Myd88^{L252P}$ animals (red). Each dot represents one mouse (n≥7; average of triplicate measurements). **(C)** Representative flow cytometry plots (left) and quantification (right) of BrdU-incorporation into germinal center B cells ($B220^+CD19^+FAS^{high}CD38^{low}$) or plasma cells ($TACI^+CD138^+$) from mice (50 weeks post immunization) that received a single injection of BrdU and were analysed 16 hours later. Each dot represents one mouse (n≥8). For Cγ1-Cre;$Myd88^{L252P}$ mice, only reporter-positive cells are shown. Increased BrdU-labeling was seen in both, germinal center B cells and plasma cells.
(D) Immunohistochemistry (left) of representative splenic sections. Scale bars: 100 µm. Germinal centers are prominent sites of proliferation. Left panel: Staining for B cells (Igκ; green), plasma cells (CD138; blue) and a proliferation marker (Ki67; red). Right panel: Quantification of $Ki67^+$ cells among plasma cells in the red pulp. Each dot represents one mouse (n≥6). Plasma cells showed little active proliferation. **(E)** Extent of somatic hypermutation in the introns downstream of JH4 in sorted $TACI^+CD138^+$ plasma cells of the indicated genotypes (in presence of $Myd88^{LP}$ also GFP reporter-positive). J558 V family genes rearranged to JH4 were amplified from genomic DNA, cloned and sequenced. One mouse per genotype was analyzed. The number of sequences analyzed is indicated in the center of the pie chart. The number of mutations is color-coded ranging from white (no mutations) to black (more than 10 mutations). IgM-positive cells from the Cγ1-Cre;$Myd88^{L252P}$ mouse carried a higher level of somatic hypermutation than IgM-positive cells from the Cγ1-Cre control mouse.

Supplementary Figure 7 (related to Main Figure 4) | Induction of $Myd88^{L252P}$ by CD19-Cre^{ERT2} restricts Myd88 mutation to a small number of B cells. **(A)** Experiment outline. CD19-Cre^{ERT2};$Myd88^{L252P}$ animals were fed once with tamoxifen in order to activate Cre-recombination in a time-restricted fashion. Mice were analyzed ten days after tamoxifen administration.
(B) Representative flow cytometry plots (left) and **(C)** quantification (right) of reporter-positive B cells ($B220^+$) and plasma cells ($TACI^+CD138^+$). Each dot represents one mouse (n≥3). As expected, only a small fraction of B cells and plasma cells contain the mutated allele and became reporter-positive after tamoxifen treatment.

Supplementary Figure 8 (related to Main Figure 4) | Activation of $Myd88^{L252P}$ in a small number of B cells leads to the development of IgM M-spikes in the serum of aged mice. CD19-Cre;$Myd88^{L252P}$ mice were analyzed at 10 and 50 weeks of age. Cγ1-Cre;$Myd88^{L252P}$ mice were analyzed 10 and 50 weeks after NP-CGG immunization. CD19-Cre^{ERT2};$Myd88^{L252P}$ animals were fed once with tamoxifen in order to activate Cre-recombination in a time-restricted fashion and the mice analyzed 1 and 70 weeks after tamoxifen administration. **(A)** Serum protein electrophoresis for the mouse strains indicated. Two of six aged Cγ1-Cre;$Myd88^{L252P}$ mice and five of five aged CD19-Cre^{ERT2};$Myd88^{L252P}$ mice showed paraprotein bands within the γ-globulin fraction (stars).
(B) Immunofixation in sera from the two Cγ1-Cre;$Myd88^{L252P}$ mice that showed M-spikes in panel A. M-spikes marked with a red star tested positive for IgM by immunofixation. **(C)** Flow cytometry plots of bone marrow-derived cells from individual aged Cγ1-Cre;$Myd88^{L252P}$ mice. GFP reporter-

positive cells (green) were further gated on B cells ($B220^{+}TACI^{-}CD138^{-}$, red) and plasma cells ($TACI^{+}CD138^{+}$, blue). **(D)** Flow cytometry plots of bone marrow derived cells from individual aged CD19-Cre^{ERT2};$Myd88^{L252P}$ animals 70 weeks after tamoxifen gavage. GFP reporter-positive cells (green) were further gated on B cells ($B220^{+}TACI^{-}CD138^{-}$, red) and plasma cells ($TACI^{+}CD138^{+}$, blue).

Supplementary Figure 9 (related to Main Figure 5) | Somatic mutations indicate intraclonal diversification in several frequently detected clonotypes 70 weeks after $Myd88^{L252P}$ induction. J558 V family genes rearranged to JH4 were amplified from genomic DNA that was isolated from sorted splenic plasma cells ($TACI^{+}CD138^{+}$), cloned and sequenced. **(A)** Controls for **Figure 5B**: Percentage of detected clonotypes in splenic IgM-positive and IgM-negative plasma cells of the indicated mouse genotypes (in presence of $Myd88^{L252P}$ also GFP reporter-positive). One mouse per genotype was analyzed. The number of sequences analyzed is shown in the center of the pie chart. The most frequently identified clonotype defined by CDR3 sequence is shown in red with the V_H gene used. The most frequently detected clonotypes in $Myd88^{L252P}$-expressing plasma cells were comparable in size to control plasma cells. **(B)** IgM and IgG expression (upper panel) and extent of somatic hypermutation in the introns downstream of JH4 (lower panel) in $TACI^{+}CD138^{+}GFP^{+}$ plasma cells from individual control mice and CD19-Cre^{ERT2};$Myd88^{L252P}$ mice 70 weeks after tamoxifen administration. Each pie chart represents one mouse. Number of mutations are color-coded ranging from white (no mutations) to black (more than 10 mutations). The overall level of somatic hypermutation in CD19-Cre^{ERT2};$Myd88^{L252P}$ mice 70 weeks after tamoxifen administration was comparable to the control mice. Plasma cells from CD19-Cre^{ERT2};$Myd88^{L252P}$ mice expressed mostly IgM. (CD19-Cre^{ERT2} control mice were tamoxifen-treated.) **(C)** Genealogical trees (reconstructed on the basis of CDR3 and downstream JH4 intron sequences) corresponding to the most frequent VDJ rearrangements per mouse as shown in the main **Figure 5B** (GFP reporter-positive plasma cells derived from bone marrow of four individual CD19-Cre^{ERT2};$Myd88^{L252P}$ mice 70 weeks after tamoxifen administration). The mouse identification number and the rearranged J558 family V gene are indicated above the tree. The number of sequences (center of pie chart) and the number of mutations is indicated (R, replacement; S, silent; I, intronic). The pattern of somatic mutations reveals intraclonal diversifications. Repeats of identical sequences may reflect clonal bursts occurring in the GC reaction **(2,3)**.

Supplementary Figure 10 (related to Main Figure 5) | Proteomic analysis confirms prevalence of IgM in M-spikes after inducible activation of $Myd88^{L252P}$. Tandem mass spectrometry of protein bands excised from Coomassie Blue-stained gels. Bands correspond to individual IgM M-spikes in the serum of five CD19-Cre^{ERT2};$Myd88^{L252P}$ mice 70 weeks after tamoxifen administration (see also **Figure 4D**). Values in the heatmap represent combined intensities (by sum) of IgH isotypes (% of the total intensity per sample). IgG subtype intensities were summed up to total isotype gamma intensity. Isotype mu was the most abundant isotype for M-spikes that had tested positive for IgM by immunofixation **(see Figure 4D)**. IgH isotypes alpha, delta and epsilon had an intensity of less than 1% in all samples (see also supplemental Table 3). The mass spectrometry proteomics data were deposited to the ProteomeXchange Consortium via the PRIDE partner repository **(1)** with the dataset identifier PXD017292 (Proteomics Identifications (PRIDE), RRID:SCR_003411).

REFERENCES FOR SUPPLEMENTARY DATA

1. Perez-Riverol Y, Csordas A, Bai J, Bernal-Llinares M, Hewapathirana S, Kundu DJ, Inuganti A, Griss J, Mayer G, Eisenacher M, et al. The PRIDE database and related tools and resources in 2019: improving support for quantification data. *Nucleic Acids Res* (2018) **47**:D442–D450. doi:10.1093/nar/gky1106

2. Tas JMJ, Mesin L, Pasqual G, Targ S, Jacobsen JT, Mano YM, Chen CS, Weill J-C, Reynaud C-A, Browne EP, et al. Visualizing antibody affinity maturation in germinal centers. *Science* (2016) **351**:1048–54. doi:10.1126/science.aad3439

3. Mesin L, Ersching J, Victora GD. Germinal Center B Cell Dynamics. *Immunity* (2016) **45**:471–482. doi:10.1016/j.immuni.2016.09.001

Supplementary Figure 1

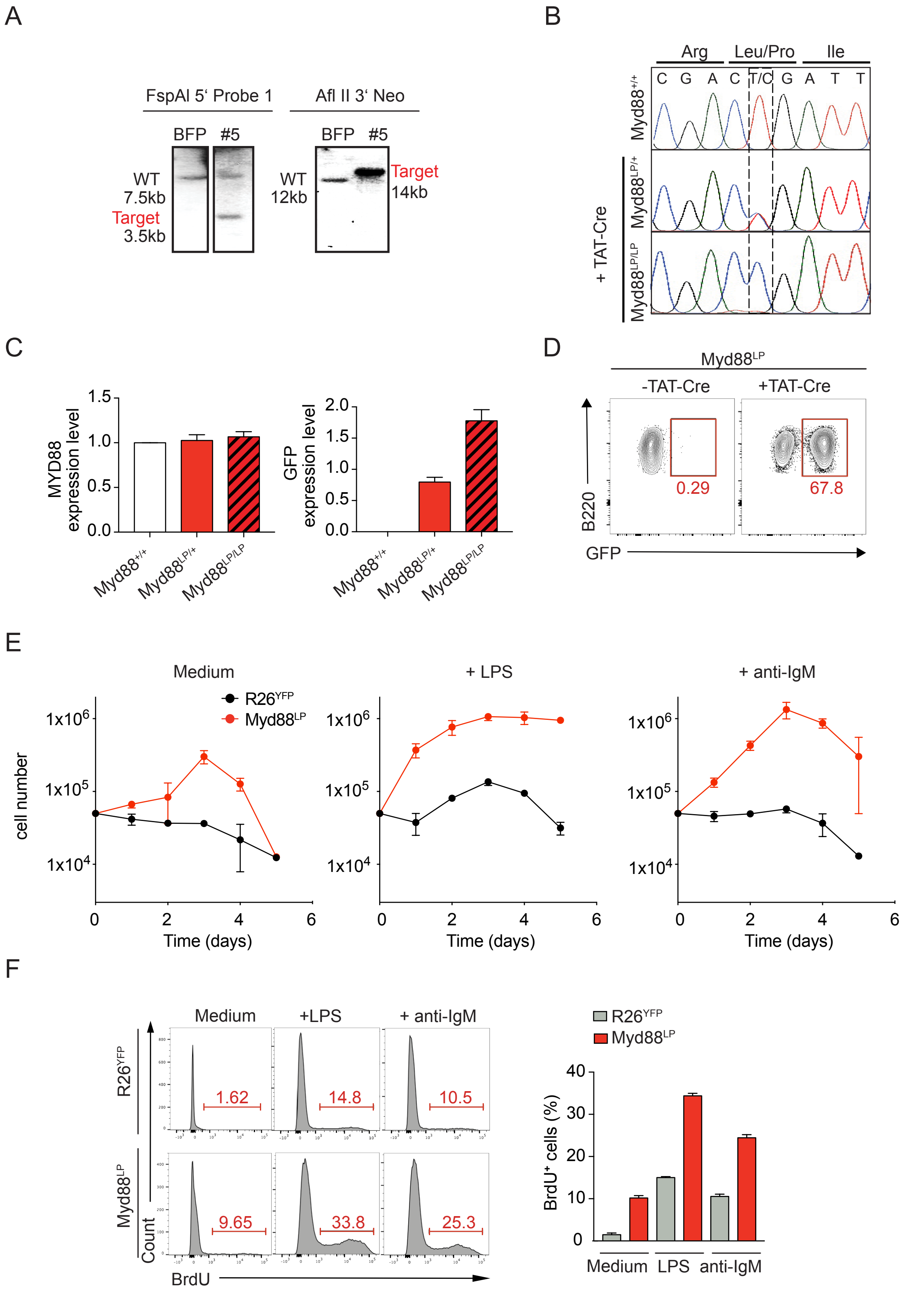

Supplementary Figure 2

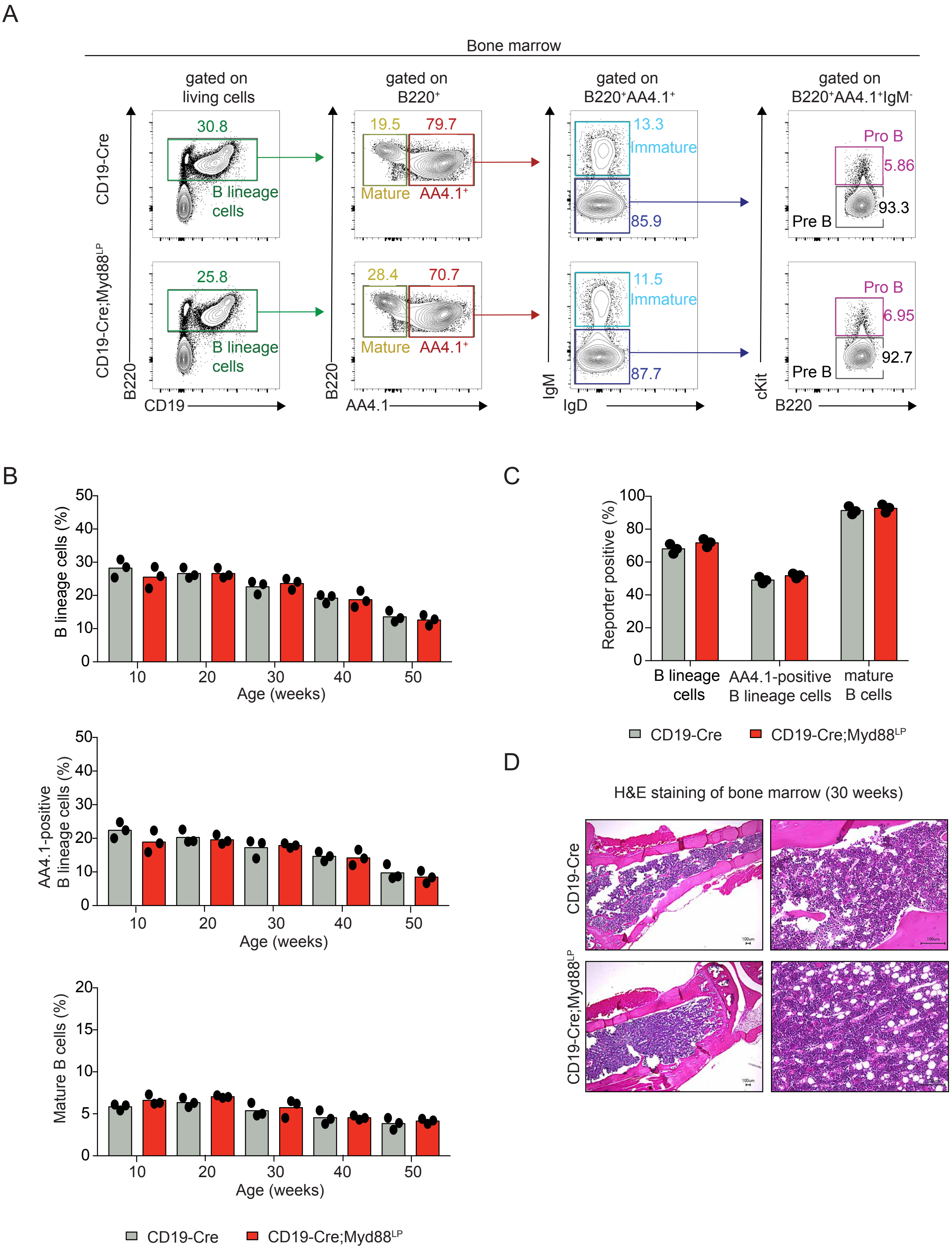

Supplementary Figure 3

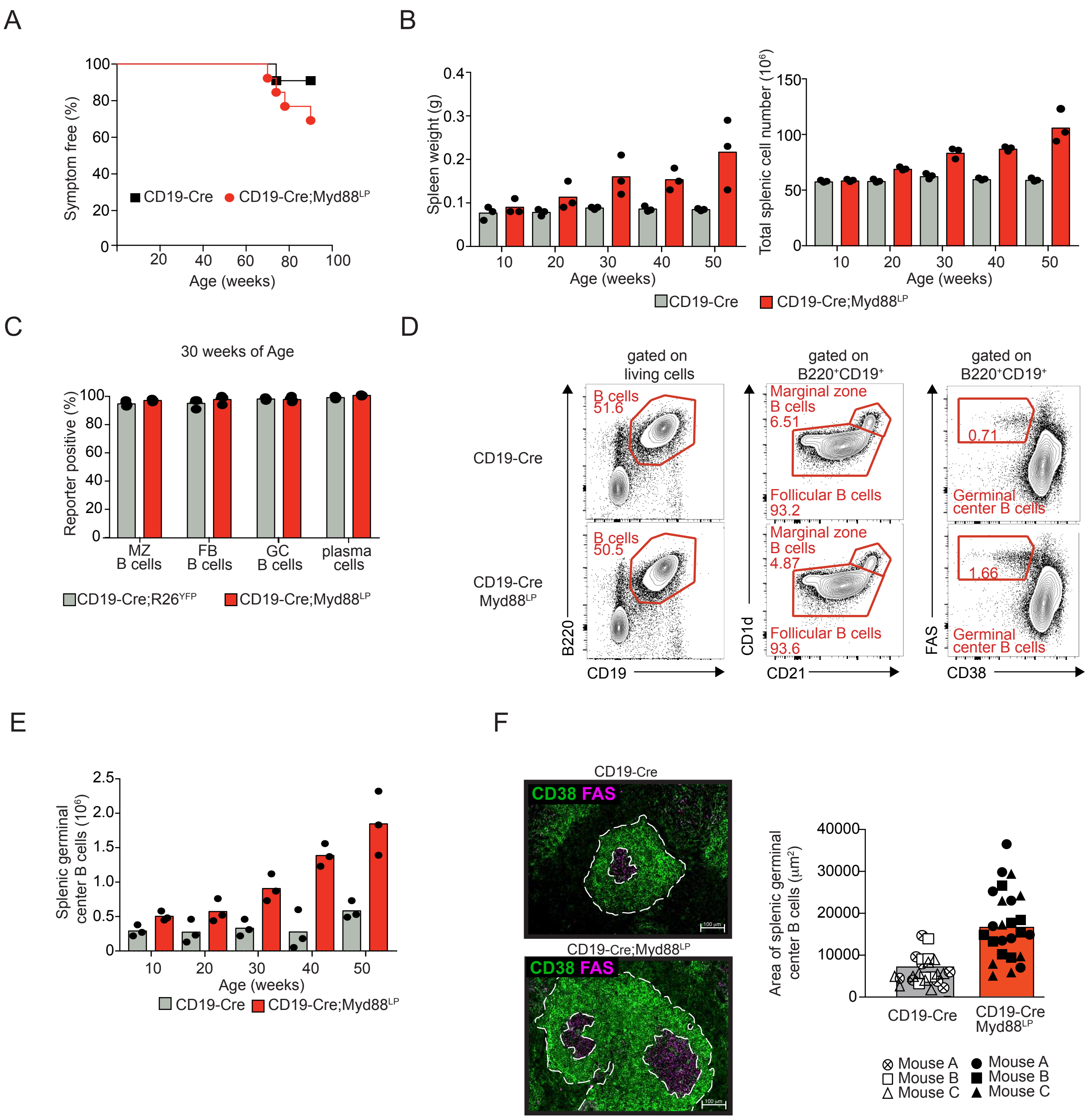

Supplementary Figure 4

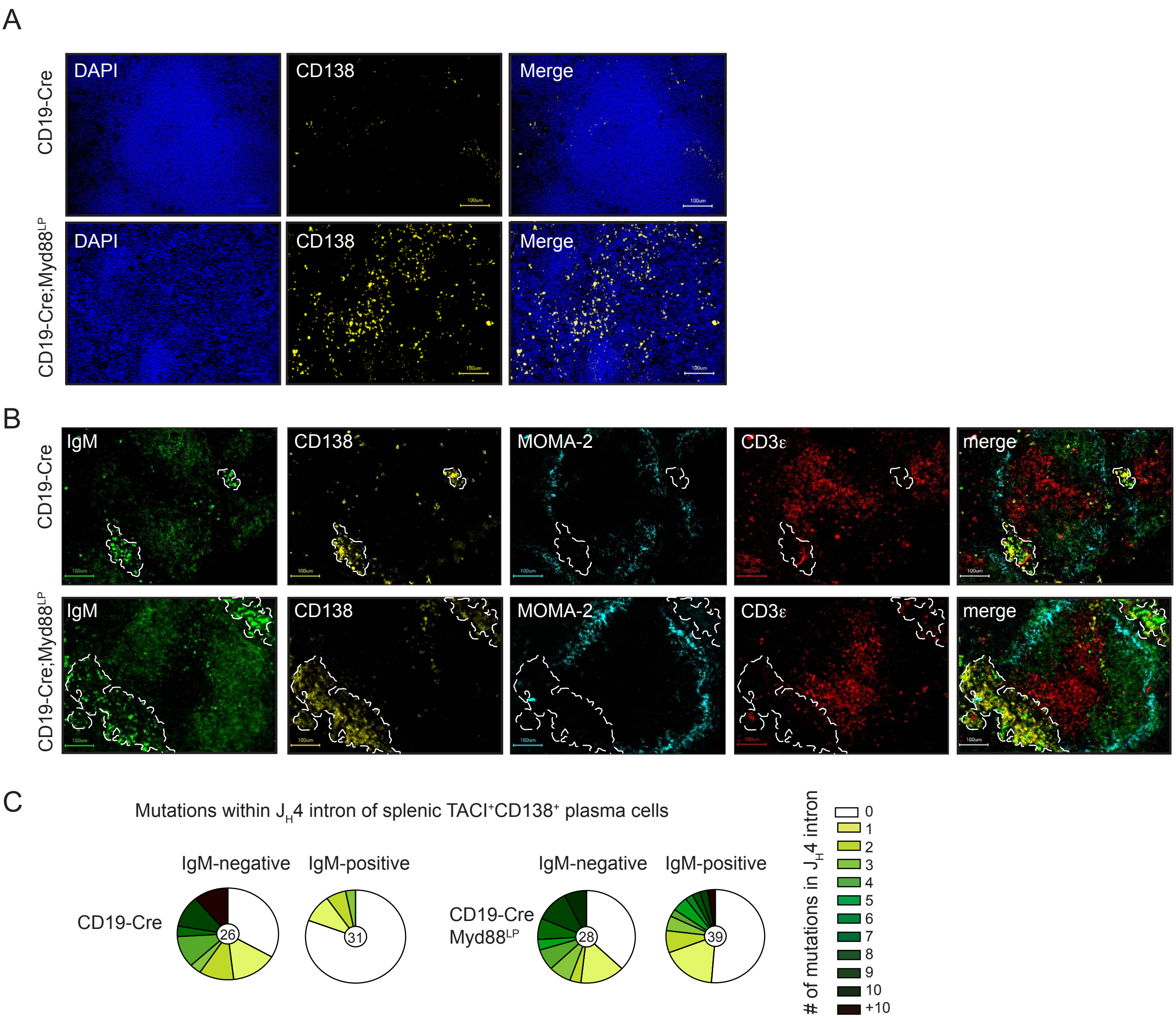

Supplementary Figure 5

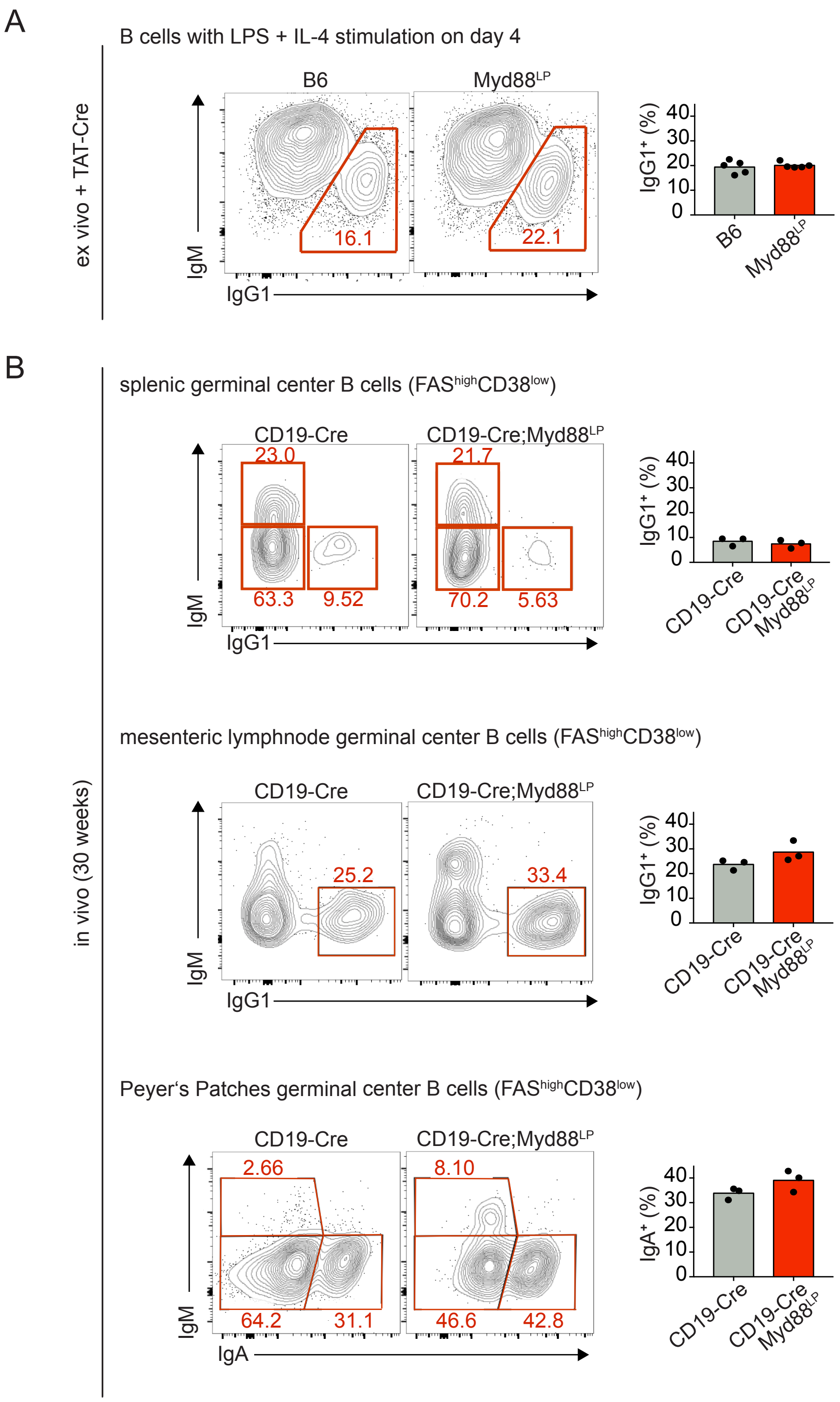

Supplementary Figure 6

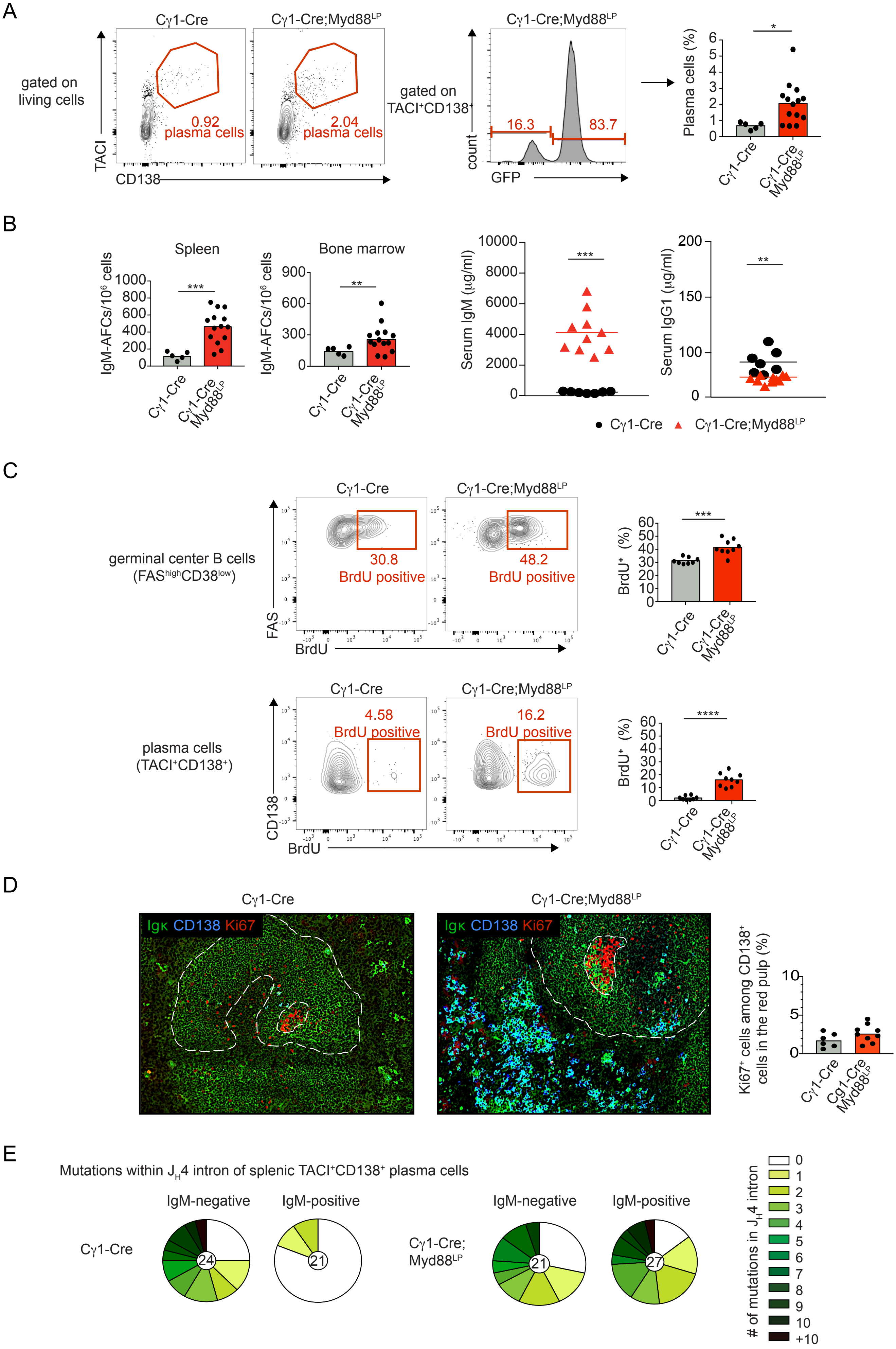

Supplementary Figure 7

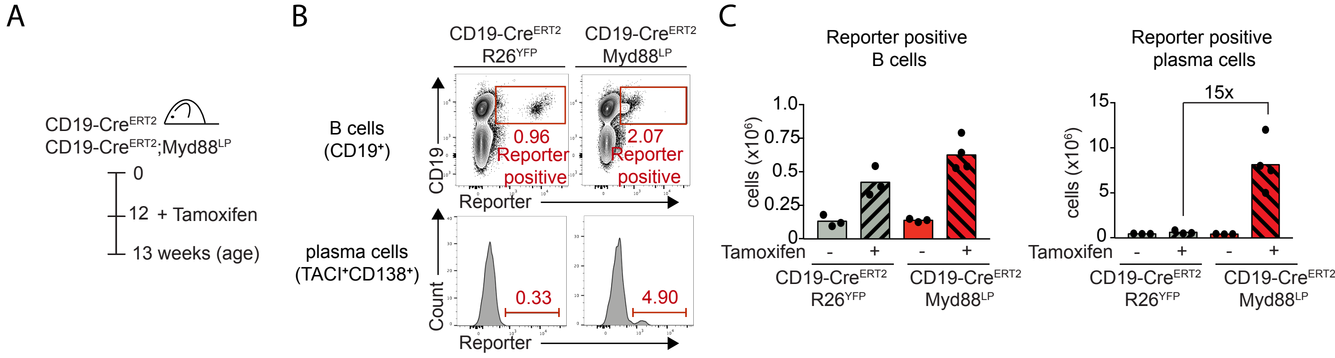

Supplementary Figure 8

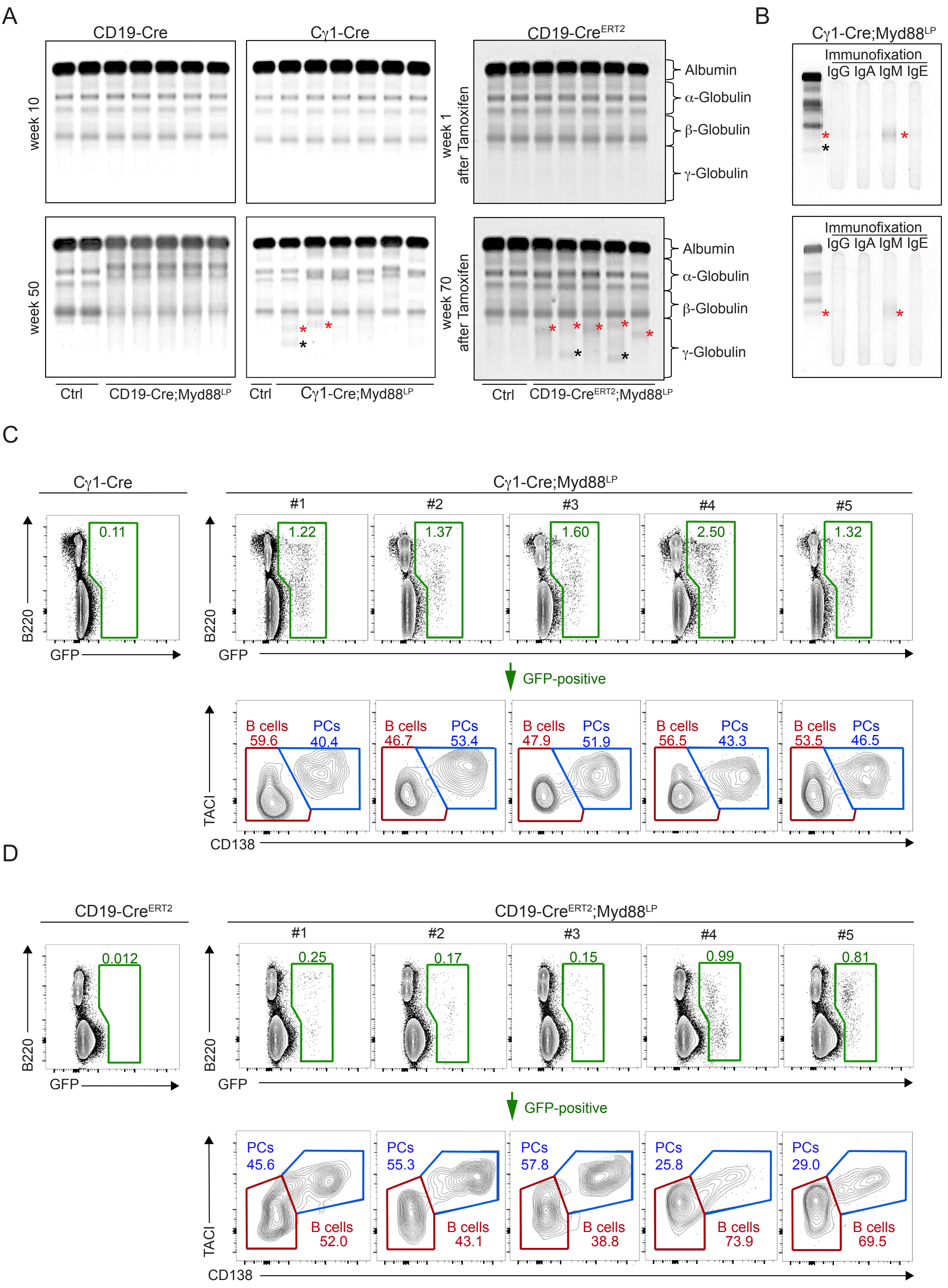

Supplementary Figure 9

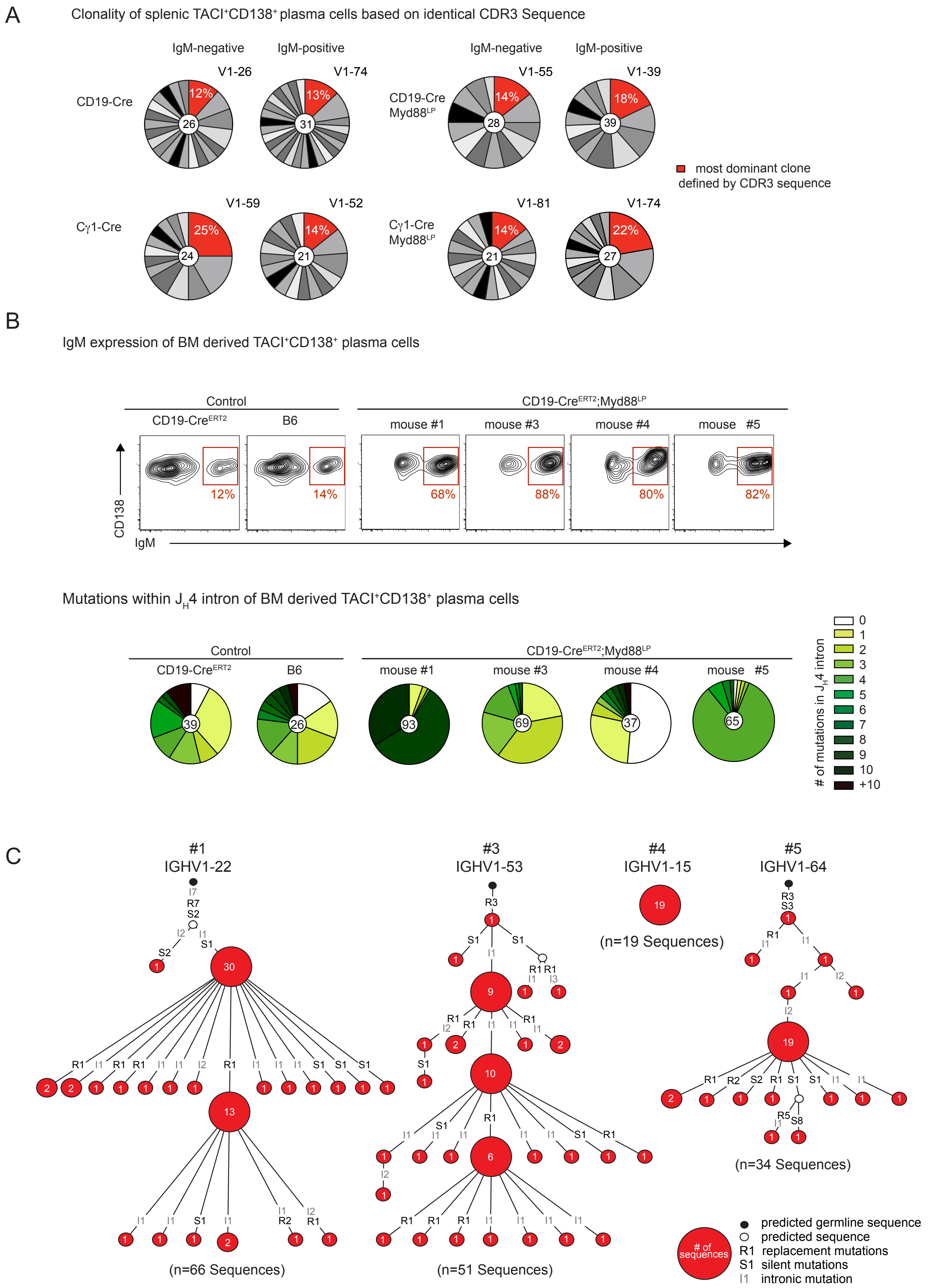

Supplementary Figure 10

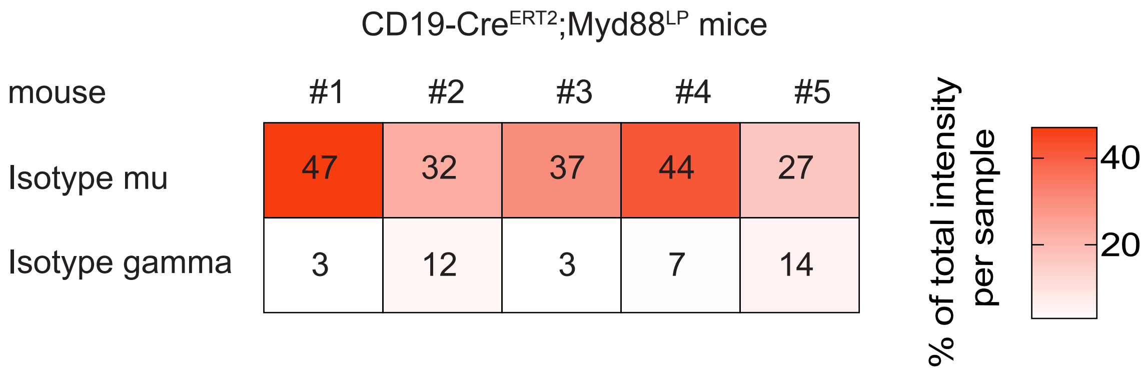

Supplement: Supplementary file 1 [file DataSheet_1.pdf]
